# Exploring the driving forces and scenario analysis for catastrophic and impoverishing health expenditures in Iran

**DOI:** 10.1186/s12913-024-10551-w

**Published:** 2024-02-26

**Authors:** Maryam Hedayati, Mohammadreza Maleki, Iravan Masoudi Asl, Ali Akbar Fazaeli, Salime Goharinezhad

**Affiliations:** 1 Department of Health Service Management, School of Health Management and Information Sciences, Iran University of Medical Sciences, No. 6, Rashid Yasemi St. Vali -e Asr Ave, P.O Box: 1996713883, Tehran, Iran; 2 Department of Health Management, Policy and Economics, Tehran University of Medical Sciences, Tehran, Iran; 3 Preventive Medicine and Public Health Research Center, Psychosocial Research Institute, Iran University of Medical Sciences, Tehran, Iran

**Keywords:** Catastrophic health care expenditure, Impoverishing, Cross-impact analysis, Driving forces, Uncertainty, Scenario based planning, Futures studies

## Abstract

**Background:**

The extent of healthcare expenditure within households stands as a crucial indicator in low and middle-income countries (LMICs). When out-of-pocket healthcare expenses surpass household income or become unduly burdensome, it serves as a significant socio-economic alarm, resulting in a reduced quality of life, a phenomenon referred to as ‘catastrophic health expenditure (CHE).’ Multiple factors can contribute to the occurrence of CHE. The study’s objective was to identify the key uncertainties and driving forces influencing CHE to develop scenarios in Iran on the horizon of 2030.

**Methods:**

This study was conducted between December 2021 and January 2023, data were collected through a literature review, and experts’ opinions were gathered via questionnaires, interviews, and expert panels. The statistical population included experts in the fields of health policy, health economics, and futures studies. Scenario Wizard software and MICMAC analysis were employed for data analysis, providing valuable insights into potential future scenarios of health expenditures in Iran.

**Results:**

Based on the results of the scoping review and semi-structured interview, 65 key factors in the fields of economics, politics, technology, social, and environmental were identified. The findings of the MICMAC analysis presented 10 key variables. Finally, six main scenario spaces are depicted using Scenario Wizard. These scenarios included catastrophic cost crises, sanction relief, selective information access, technological ambiguity, induced demand management, and incremental reforms.

**Conclusions:**

Each of the six drawn scenarios provides images of the future of health expenditure in Iranian households on the horizon of 2030. The worst-case scenario from all scenarios was scenario one, with the most probable and critical features to derive Iran’s health expenditures. The current study is a valuable addition to the literature depicting the key drivers that all developing nations can consider to decrease exposing households to catastrophic and impoverishing health expenditures.

**Supplementary Information:**

The online version contains supplementary material available at 10.1186/s12913-024-10551-w.

## Introduction

Universal health coverage (UHC) is a fundamental goal embedded within the Sustainable Development Goals (SDGs), reflecting the overarching aim of nations to establish sustainable and equitable healthcare systems. The global commitment to achieving UHC by 2030, as endorsed by countries worldwide through the SDGs, emphasizes the World Health Organization’s (WHO) definition of UHC, ensuring access to essential public health services, prevention, treatment, rehabilitation, and palliative care with the highest quality and safety standards, while mitigating financial burdens on individuals and communities [1]. Within the broader context of the third SDG, an essential objective is to shield households from financial hardships and promote fairness in financial contributions, aiming to protect families from healthcare-related expenses. The healthcare system’s central mission includes minimizing vulnerabilities to catastrophic and impoverishing health expenditures, recognizing the pivotal role of financial equity [2].

In many developing countries, including Iran, health financing predominantly relies on out-of-pocket payments (OOPs), wherein households allocate a portion of their income to healthcare expenses. However, the simplicity of this approach is accompanied by inefficiency and risk, particularly due to the lack of a risk pooling mechanism [3]. Equity in healthcare systems is contingent on the alignment of service utilization with population needs (equity in delivery) and proportional financial contributions to individuals’ ability to pay (equity in financing) [4].

When patients are unable to access necessary treatments and services, it not only affects their ability to pay for essentials such as food and housing but also diminishes their overall quality of life. This outcome often results from an imbalanced OOPs mechanism [5]. The 2019 Iran National Health Accounts report highlighted a concerning upward trajectory, with out-of-pocket (OOP) expenditures constituting 37% of total health spending in 2019, surpassing the set target of 30% outlined in consecutive five-year development plans [6–8]. This reliance on OOP payments has contributed to widespread financial hardships globally, affecting over 930 million individuals seeking healthcare and leading to an estimated 100 million people falling into poverty annually [9].

The substantial reliance on OOP payments in Iran, accounting for approximately 39.49% of current health expenditures, as reported by the World Health Organization’s Eastern Mediterranean Regional Office (EMRO), has resulted in millions of individuals foregoing necessary healthcare, leaving critical medical needs unmet [10, 11]. The burden of CHE is a critical concern within the Iranian healthcare system, as evidenced by a series of systematic reviews and meta-analyses, Aryankhesal et al. (2018) conducted a comprehensive study, revealing that approximately 7.5% of households in Iran faced CHE [12]. This finding was echoed in subsequent research by Rezaei et al. (2019), who reported an average prevalence of 7% of households experiencing CHE [13]. Furthermore, Doshmangir et al.‘s meta-analysis in 2020 provided a nuanced perspective, indicating that the population-level rate of CHE in Iran was 4.7%, with a notable impact on 25.3% of households when assessed across various diseases [14].

An investigation into catastrophic and impoverishing health expenditures, two pivotal indicators of financial protection within the context of the Sustainable Development Goals (SDGs), reveals that the Iranian healthcare system is progressing toward achieving UHC. As we strive towards achieving UHC by 2030, understanding and addressing the factors contributing to CHE become paramount. This study endeavors to contribute to this imperative by employing foresight science and cross-impact analysis to forecast future trends in catastrophic health expenditures and propose strategic interventions. Through an exploration of potential scenarios, the research aims to inform policymakers and healthcare stakeholders in developing a resilient and equitable healthcare framework for the future.

## Method

### Study sesign

This research adopts an exploratory approach, combining elements of both quantitative and qualitative studies [15]. The scenario development unfolds in three key stages: the identification of driving forces or key influences, the recognition of uncertainties, and the formulation of various narrative scenarios, ultimately leading to scenario mapping. The study was conducted between December 2021 and September 2022.

### Structural analysis perspective

Drawing from cross-impact analysis (CIA) utilizing MICMAC software, the study aims to derive optimal scenarios for mapping the future of catastrophic and impoverishing health expenditures in Iran (Supplementary 1 and 2).

### Qualitative phase

In the qualitative phase, the research commenced with a scoping review and semi-structured interviews with key informants. This approach allowed for a comprehensive analysis of the problem, resulting in the compilation of an extensive list of trends, driving forces, variables, and uncertainties with the most substantial impact on the central scenario issue.

### Synthesis phase

Subsequently, the research entered a synthesis phase with the aim of distilling a limited number of driving forces and uncertainties from those identified during the exploratory phase. The key drivers were categorized using a Likert questionnaire and Porter’s five forces framework, which includes social, technical, economic, environmental, and political (STEEP) indicators within the macro-environment that influence Catastrophic Health Expenditure (CHE) in Iran [16].

### Prioritization of key drivers

The next step involved the prioritization of these key drivers through the application of the Wilson matrix, considering both the drivers’ future uncertainty and impact. This systematic evaluation method assessed the significance of the drivers.

### Cross-impact analysis

Subsequently, the study delved into Cross-Impact Analysis, a widely utilized quantitative method in futures studies. This approach systematically described all potential modes of interaction between variables and assessed the strength of these interactions [17].

### Statistical surveys and software

Statistical surveys were incorporated into the model framework, supported by specialized software, including MicMac and Scenario Wizard. This enabled the pinpointing of the most influential factors in terms of importance and uncertainty.

### Scenario formulation

With this foundation in place, the research delineated possible scenarios. The creation of scenarios based on cross-impact analysis (CIA) typically involves four step which are described in detail [18, 19].

### Step1: Identifying key variables or driving forces

In this step, the analysis’s scope, the scenario field, and the overall work framework are delineated. Concurrently, the gathering of initial data and information is undertaken [20]. The results of this step lead to the identification of variables influencing exposure to catastrophic and impoverishing health expenditures in Iran. These variables are informed by a Scoping review and semi-structured interviews, elucidated further below.

## Scoping review

A scoping review was conducted following the Joanna Briggs Institute method and in accordance with the PRISMA-ScR checklist. The framework of Arksey and O’Malley [21] was used for performing scoping reviews in five stages as follow:

### Stage 1: Identifying research questions

The primary question guiding the scoping review was: What are the main determinants, trends, driving forces, and key uncertainties influencing household exposure to catastrophic and impoverishing healthcare expenditures in Iran in the future?

### Stage 2: Identifying relevant studies

To identify pertinent studies, we conducted a comprehensive search across various databases, including PubMed, Scopus, Web of Science, and four Persian databases (Scientific Information Database [SID], IranMedex, IranDoc, Magiran), as well as Google Scholar. Additionally, we explored the gray literature available in the virtual libraries of relevant organizations such as the World Health Organization (WHO), the World Bank, and the Ministry of Health and Medical Education (MOHME). The search, conducted from 2000 to December 30, 2021, was extended to include references from selected studies. The search strategy for the PubMed database is outlined below and was adjusted for other databases (refer to Supplementary 3). The complete results of the search are presented in the final review and illustrated in a PRISMA-SCR flow diagram [22] (Supplementary 4). The PRISMA-SCR checklist has been developed in Supplementary 5.

### Stage 3: Study selection

All studies obtained in the search stage were transferred to an EndNote. All studies providing information on catastrophic and impoverishing healthcare expenditures and determinants in Iran were eligible for inclusion. Two authors (MH, SG) independently screened and reviewed potentially relevant articles.

### Stage 4: Charting the data

A charting form, developed by two reviewers (MH, SG), facilitated data extraction from selected studies, covering general features and key results.

### Stage 5: Collating, summarizing, and reporting results

Data analysis was conducted based on the data extraction form, and variables were extracted for further investigation.

## Semi-structured interviews

As part of the cross-impact analysis’s first step, semi-structured interviews were conducted with stakeholders to identify comprehensive factors influencing catastrophic and impoverishing healthcare expenditures in Iran. The focus was on confirming factors identified in the previous step.

### Sampling and inclusion criteria

Purposive and snowball sampling identified stakeholders with an in-depth understanding and rich information about the study’s focus, including health policymakers, managers, and researchers from various disciplines.

### Data collection

Ten in-depth interviews were conducted by MH between March and April 2022, with informed consent obtained from participants. An interview guide (Supplementary 6), developed through literature review and meetings, clarified the research’s main objective.

### Data analysis

Thematic content analysis was employed, and emerging themes were identified and consolidated iteratively. The analysis utilized MAXQDA 2020 qualitative software to identify themes.

### The pilot test methods

With the purpose of conducting the pilot testing of this semi structured interview, the researcher has put a lot of effort into ensuring the important things related to it including the participants, setting, research instrument, and three procedures of the interview session (1. pre-interview stage, 2. during the interview stage and 3. post-interview stage).

### Trustworthiness

To enhance dependability, the first author primarily handled data collection, ensuring consistency in queries. Credibility was strengthened through collaborative data analysis by two authors. Participants verified interview texts and extracted codes to enhance transferability.

### Step 2: Finalize, categorize, and prioritize key variables

In this step, the research team achieved consensus to create a definitive list of key variables, ensuring each variable’s clarity and understanding among all respondents. A self-administered questionnaire was employed for finalizing and categorizing these variables, using a seven-point Likert scale (1– strongly disagree to 7– strongly agree). The Likert scale gauged respondents’ agreement or disagreement with each driver related to catastrophic and impoverishing health expenditure. Additionally, the STEEP framework was adopted in the questionnaire to categorize key drivers into social, technological, environmental, economic, and political/legal dimensions [16]. To enable nationwide expert participation, communication was established through email and online meetings. Participants were informed of the voluntary nature of their participation, and their anonymity and personal information confidentiality were assured. The informed consent form was read and signed by participants.

Subsequently, the Wilson matrix was employed to evaluate and prioritize the impact and uncertainty of each scenario driver on the future. This matrix ranks factors based on potential impact and probability, identifying critical uncertainties that form the basis of scenario construction [23]. To pinpoint the most critical scenario elements, experts participated in a two-round Delphi survey, evaluating each factor (scenario driver) based on its potential impact on the objective and associated uncertainty. A questionnaire was distributed to twenty experts to gather their input, with two rounds ensuring comprehensive feedback and assessment. In the resulting matrix, factors with high priority are highlighted in light blue on the upper right side, those with medium priority are depicted in white, and factors with low priority are marked in green on the lower left side.

### Step 3: Identification of key variables through cross-impact analysis (CIA)

In this phase, the interrelationships among existing variables are systematically described using the Cross-Impact Analysis (CIA) method, a popular tool in futures studies [17]. CIA, considered a soft-systems tool, can be qualitative or quantitative. In this study, a qualitative approach based on structural analysis was employed [24].

CIA Steps:

Variable definition:

Variables affecting the future exposure to Catastrophic Health Expenditure (CHE) were derived from the preceding steps.

Interactions analysis:

Variables entered an interaction matrix, and relationships were determined by experts.

Variables were weighted (0 to 3) based on the degree of influence.

This step involved the collection of variables, describing their relationships, and identifying key variables.

Chart analysis and visual representation:

The roles of variables were identified, and an influence–dependence value was assigned for interpretation.

A two-dimensional map with vertical and horizontal axes represented influence and dependence.

Chart zones:

Determinant/Influential Factors: Located in the northwest quarter, these factors are crucial inputs with a significant impact on the system.

Intermediate/Key Variables: Situated in the northeast quarter, these are both influential and dependent, divided into stake and target variables.

Dependent/Output Variables: Found in the southeast position, these are highly dependent on influential factors.

Autonomous/Excluded Variables: In the southwest quarter, these have little influence and dependence.

Clustered/Neuter Variables: Positioned in the border areas, these variables are likely to join other variables.

Selection of key variables:

Variables in determinant positions, with high influence and dependence, were identified as key and critical.

This structured approach using the CIA provides a comprehensive understanding of key variables influencing the future of catastrophic and impoverishing health expenditures. The systematic assessment aids in decision-making for robust future scenarios. (Supplementary 7).

### Step 4: Scenario development through cross-impact balance (CIB) analysis

In the final stage, we employed the Cross-Impact Balance (CIB) analysis method for scenario development due to its qualitative orientation, aligning well with expert judgments and addressing data constraints [25]. CIB offers a structured approach to eliciting expert knowledge about the strength and nature of relationships within a system, making it ideal for identifying qualitative scenarios.

Steps of the CIB process:

Expert panel assembly:

A panel with rich knowledge about key variables convenes.

Descriptor compilation:

A list of relevant system factors, known as descriptors, is compiled. Key factors are extracted from the MICMAC technique.

Qualitative alternatives definition:

Sets of qualitative alternatives (variants) defining possible states of the descriptors are determined.

Example:

a. X_1_ {x_a_, x_b_, x_c_}.

b. X_2_ {x_x_, x_y_, x_z_}.

c. X_3_ {x_i_, x_j_}.

X_n_ {x_1_ … x_n_}.

Impact Evaluation:

The expert panel determines the influence–dependence of key factors using cross-impact judgments on a qualitative scale.

Judgment scale:

+ 3: strongly promoting influence.

+ 2: promoting influence.

+ 1: weakly promoting influence.

0: no influence.

−1: weakly restricting influence.

−2: restricting influence.

−3: strongly restricting influence.

Cross-impact matrix is drawn using Scenario Wizard software.

Consistent scenario calculation:

Consistent configurations of the impact network (“consistent scenarios”) are calculated through the CIB algorithm.

Inconsistency coefficients (0–2) identify strong and weak scenarios.

Experts explain narrative expressions for each scenario.

In summary, our study design and method employed a structured and systematic approach to scenario development, relying on expert knowledge and qualitative judgment. The success of this approach hinges on the expertise of the panel, accuracy in cross-impact judgments, and the validity of the scenarios created.

## Results

### Identifying key variables based on cross-impact analysis

To determine the most important or key variables affecting catastrophic and impoverishing healthcare expenditures in Iran a scoping review and interviewing experts were conducted. The reviewers had concurrence to include 101 articles that assessed the determinants of CHE in Iran in the final review analysis. supplementary 4 illustrates the PRISMA-ScR [22] flowchart. The synthesis results are presented in supplementary 8. As the supplementary 9 depicts, 110 key variables were collected through reviewing related previous studies. Due to the diversity of determinants in terms of number and nature, they were divided into six categories. The categories included socioeconomics characteristics of the household, demographic characteristics of the household, vulnerable persons in the household, Health care utilization by household members, health expenditure indicators, and macroeconomic indicators. Through interviewing experts (including academic and administrative experts in the Iranian health system) 15 other variables affecting Iranian households with catastrophic and impoverishing health expenditures were identified that were divide to five categories (supplementary 10).

In the next step, based on the 20 experts’ opinion and by the seven-point Likert scale some factors were removed, merged, or modified, and finally, 65 factors affecting CHE were obtained. Afterward, the finalized variables were categorized by using the STEEP framework and prioritized by the using the scenario tool, i.e. Wilson matrix to assess the degree of uncertainty and impact of the key variables by a panel of specialists.

The variables assigned with a high impact & high uncertainty that classified as critical uncertainties were “Increasing consumption of expensive high-tech health care services”, “Informal payments or under-the-counter payment”, “Induced demand (consumer or supplier(”, “budget deficit of the health system”, “The increase in the price of medicine and medical devices due to the increase in the exchange rate and the removal of the preferential currency subsidy”, “Lack of reliable and transparent electronic information systems”, “Conflict of interests of Iran’s health system decision-makers”, “Lack of implementation of family physician and referral system in the whole country”, “Economic sanctions against Iran”, and “Inflation rate in health sector” (supplementary 11). Supplementary 12 highlights the Wilson matrix created to evaluate and prioritize the impact and uncertainty of each scenario driver against two dimensions: potential impact and uncertainty.

In the last step, the final variables were adapted in the form of a 29 × 29 cross-impact matrix. The cross-impact matrix was finalized in an expert panel of 15 experts in the field to determine the impact of these key factors. Supplementary 13 show the average scores provided by Individuals.

The validation of the structural analysis based on MICMAC was conducted with domain experts [26]. After collecting the data from the expert panel, using the MICMAC technique and cross-impact analysis, the effects of factors were calculated directly and indirectly. The amount of matrix filtration was 82, which indicates that 25.80% of the variables have an influence on each other. To summarize, of 217 matrix-based relationships, 624 had no relationship, 180 (82.94%) had weak relationships, 30 (13.82%) had moderate relationships, and 7 (3.22%) had strong relationships with each other (supplementary 14).

The direct and the points of each factor are shown in the column and the matrix row. Supplementary 15 shows the direct effects of factors and supplementary 16 shows the indirect effects of factors.

The information from the cross-impact matrix based on matrix of direct influence (MDI) indicates that most of the variables have an important role in exposing households with catastrophic and impoverishing health expenditures in future, but only some of them have maximum influence on the system and are known as key variables of the system.

The final matrix results in five main zones including input, intermediate/key, clustered, resultant, and excluded variables. In supplementary 17 the variables are distributed in five zones and each of the zones has a specific character. For example, “economic sanctions against Iran” and “high inflation rate in health sector” and “conflict of interests of Iran’s health system decision-makers” are identified as input/influential variables; “budget deficit of the health system” is intermediate/key variables; “lack of implementation of family physician and referral” or “lack of reliable and transparent electronic information systems” are neuter variables; and “the increase in the price of medicine and medical devices” and “increasing consumption of expensive high-tech health care services” are clustered/independent variables, and “Lack of financial protection” is dependent variable.

**Fig. 1:**
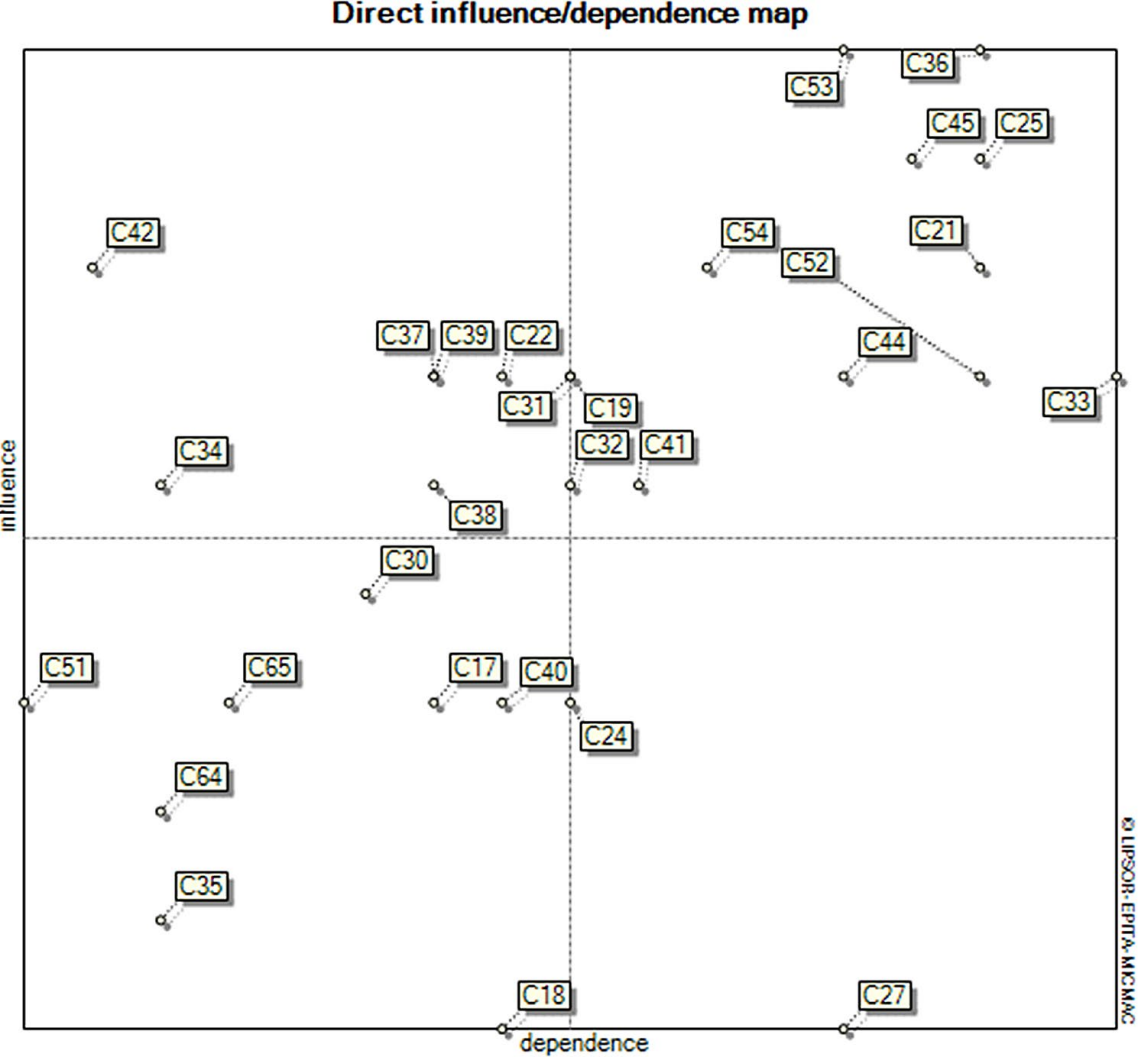
Influence and dependence of variables

Fig. 1 indicates that the intermediate variables, which are located in the northeastern part of the map, are considered as important variables because of their high degree of direct influence. In other words, intermediate variables always have a high degree of influence and usually high dependence. According to Fig. 1 those variables with the highest degree of direct influence on the CHE in Iran are located in northeastern part of the plotted map. Key variables are the most important and influential variables in the future of Iran’s catastrophic and impoverishing health expenditure.

**Fig. 2:**
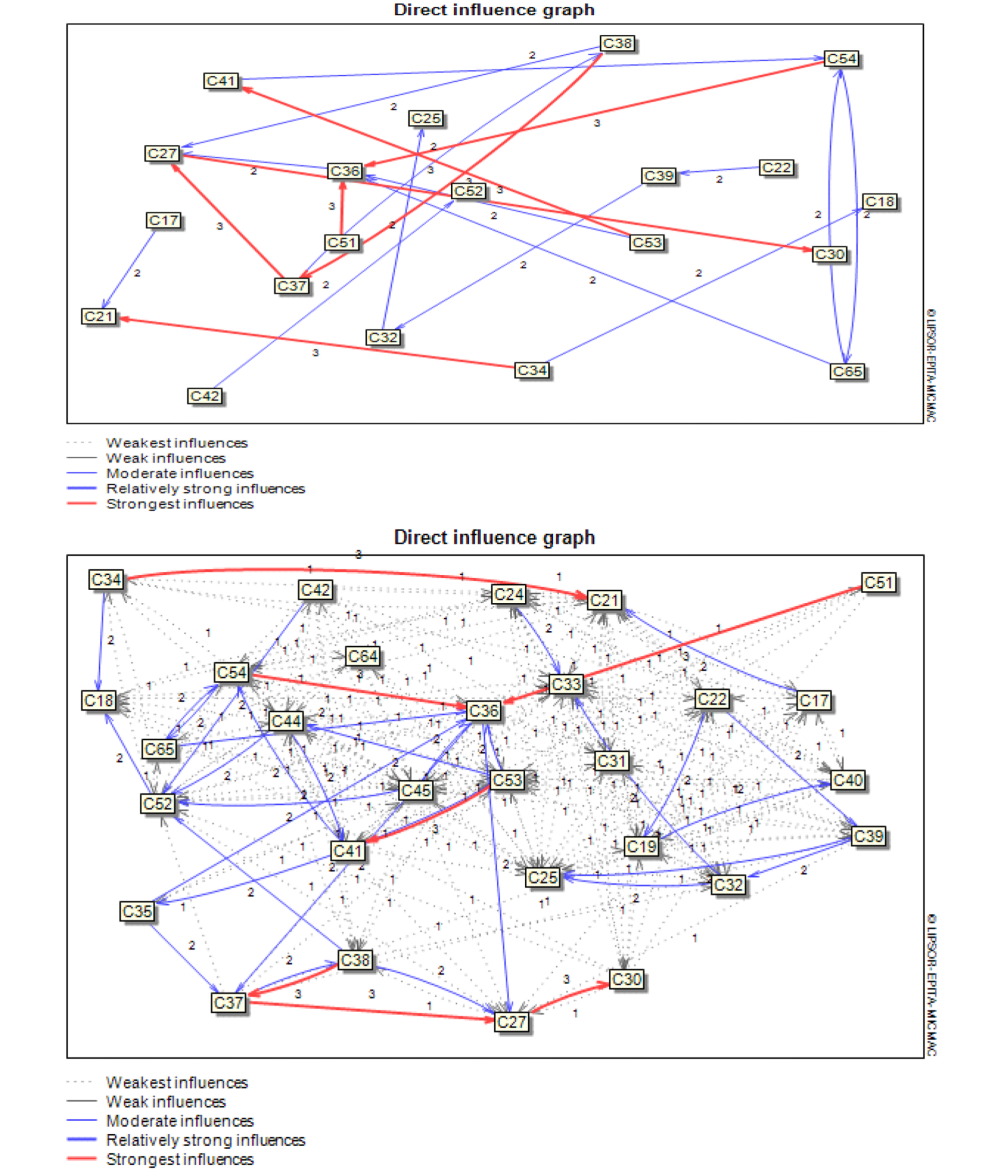
Spatial structure of direct indicators with 10% and 100% rate

After determining the status of each factor affecting the catastrophic and impoverishing health expenditures, the relationships of these factors were investigated in the MicMac software and the relationships of the effects of factors were shown directly and indirectly in the figures below. Fig. 2 indicates that the spatial structure of direct drivers of Iran’s catastrophic and impoverishing health expenditure at a 10% rate are constructed by indicators. In other words, these have a high degree of influence on some indicators and may have a high dependence on others. The spatial structure of direct drivers of catastrophic and impoverishing health expenditure with a 100% rate contains all kinds of relationships (potential, strong, moderate, weak, and none). Those are the most important indicators in the constructed spatial structure of the catastrophic and impoverishing health expenditure in Iran.

### Building consistent scenarios based on CIB

In this step, the aim was to provide an appropriate structure of scenario assumptions to prevent contradictions and inconsistencies in the way each descriptor influenced the origin and purpose of the study. The CIB matrix provided during this process required variables to be defined in such a way that no variable of the same descriptor would be preferred over another to maintain the inherent consistency of the matrix [25]. The focus was on building possible scenarios for the study’s ten key variables identified using the cross-impact algorithm. The key descriptors, with coded rules in the form of the CIB method, were once again arranged and distributed among the members of the expert panel for review. A 30 × 30 matrix was formulated to examine how a change in any of the descriptor variables could impact catastrophic and impoverishment health expenditures in Iran. Scenarios were developed based on forthcoming judgments, relationships, and interactions of variables and structured processes. Once a list of the most relevant system factors (“descriptors”) was compiled, it was necessary to define a set of qualitative alternatives (variants) that characterized possible states of the descriptors and included various states of catastrophic and impoverishment health expenditures in the future of Iran. In fact, these states could occur in the health system and be considered as strategic drivers for the future of catastrophic health expenditures on a national scale. Table 1 illustrates the study’s descriptors along with their respective variables that characterize the possible state of the descriptors.

**Table 1:** Possible states of descriptors

Descriptor	Variants		
A. Budget deficit of the health system	A_1_. Increasing the budget of the health system	A_2_. The stability of the budget of the health system	A_3_. Reducing the budget of the health system
B. Economic sanctions against Iran	B_1_. Lifting Economic Sanctions on Iran	B_2_. The effect of sanctions decreases	B_3_. sanctions have more severe consequences.
C. Informal payments or under-the-counter payment	C_1_. Such payments are impossible (eradicate informal payments).	C_2_. Medical staff are willing to receive an under-the-counter payment before treatment	C_3_.Informal payments are deeply ingrained in the health care sector
D. Conflict of interests of Iran’s health system decision-makers	D_1_. No conflict of interest	D_2_. Moderate conflict of interest	D_3_.Severe conflict of interest
E. Increasing consumption of expensive high-tech health care services	E_1_. Decreasing consumption of expensive high-tech health care services due to decreased access to advanced medical technologies and decrease in willingness to pay	E_2_. Consistency of consumption due to no change in willingness to pay for advanced technologies without changing access to them	E_3_. Increasing consumption of expensive high-tech health care services due to increased access to advanced medical technologies and increase in willingness to pay
F. Inflation rate in health sector	F_1_. The inflation rate of the health system is reduced	F_2_. The inflation rate of the health system remains constant	F_3_. The inflation rate in the health sector increases at a rate twice higher as the prevailing inflation rates
G. Induced demand (consumer or supplier)	G_1_. The decrease in induced demand	G_2_. The absence of substantial change in induced demand	G_3_. The increase in consumer surplus due to induced demand
H. Lack of reliable and transparent electronic information systems in the health sector	H_1_. Developing integrated health information systems in the health sector	H_2_. Access to integrated and validated information for specific authorities	H_3_. Lack of access to up-to-date and integrated information systems.
I. Lack of implementation of family physician plan (FPP)and referral system in the whole country	I_1_. Complete and successful implementation and performance of FPP and referral system in Iran	I_2_. Step-by-step implementation of the FPP and referral system in provinces	I_3_. The FPP and referral system are not implemented in Iran.
J. The increase in the price of medicines and medical devices due to the increase in the exchange rate and the elimination of the preferential currency subsidy	J_1_. Not to eliminate preferential currency subsidies and expensive drugs were included in the basic health insurance benefit packages.	J_2_. A step-by-step approach to eliminating preferential currency subsidies and the price of medicines and medical devices is gradually increasing.	J_3_. Eliminate preferential currency subsidies and Sudden increase in medicines and medical devices prices.

In the next phase, judgments about the impact of state x of descriptor X on state y of descriptor Y were made, based on expert panel. Only direct influences were accounted for in these judgments. This procedure results in a cross-impact matrix in Scenario Wizard software, with 30 possible states for 10 key variables (descriptors) that influence exposing Iranian households to catastrophic health expenditures and impoverishment. In order to check consistent scenarios, the indicators of the Consistency value and total impact score were used. From this number of possible states, 59,049 possible combining scenarios were extracted (from 3 × 3 × 3 × 3 × 3 × 3 × 3 × 3 × 3 × 3). The scenarios were presented in Scenario Wizard, including 6 scenarios with strong consistencies, 154 scenarios with weak consistencies, and 58 inconsistent scenarios. The results demonstrate that 6 scenarios are highly likely to occur in the future of catastrophic and impoverishing health expenditures in Iran, focusing on Reducing exposure to CHE. The six-strong consistent scenarios are characterized according to specific features; among these six scenarios, the first scenario is driving scenario, the second and third scenarios have an intermediate status, and the fourth, fifth, and sixth scenario has a critical and undesirable status and is inappropriate for Iranian households to exposed with catastrophic and impoverishing health expenditures. In Table 2 scenarios with strong consistencies and the possible states of each key variable in every scenario are indicated.

**Table 2:**
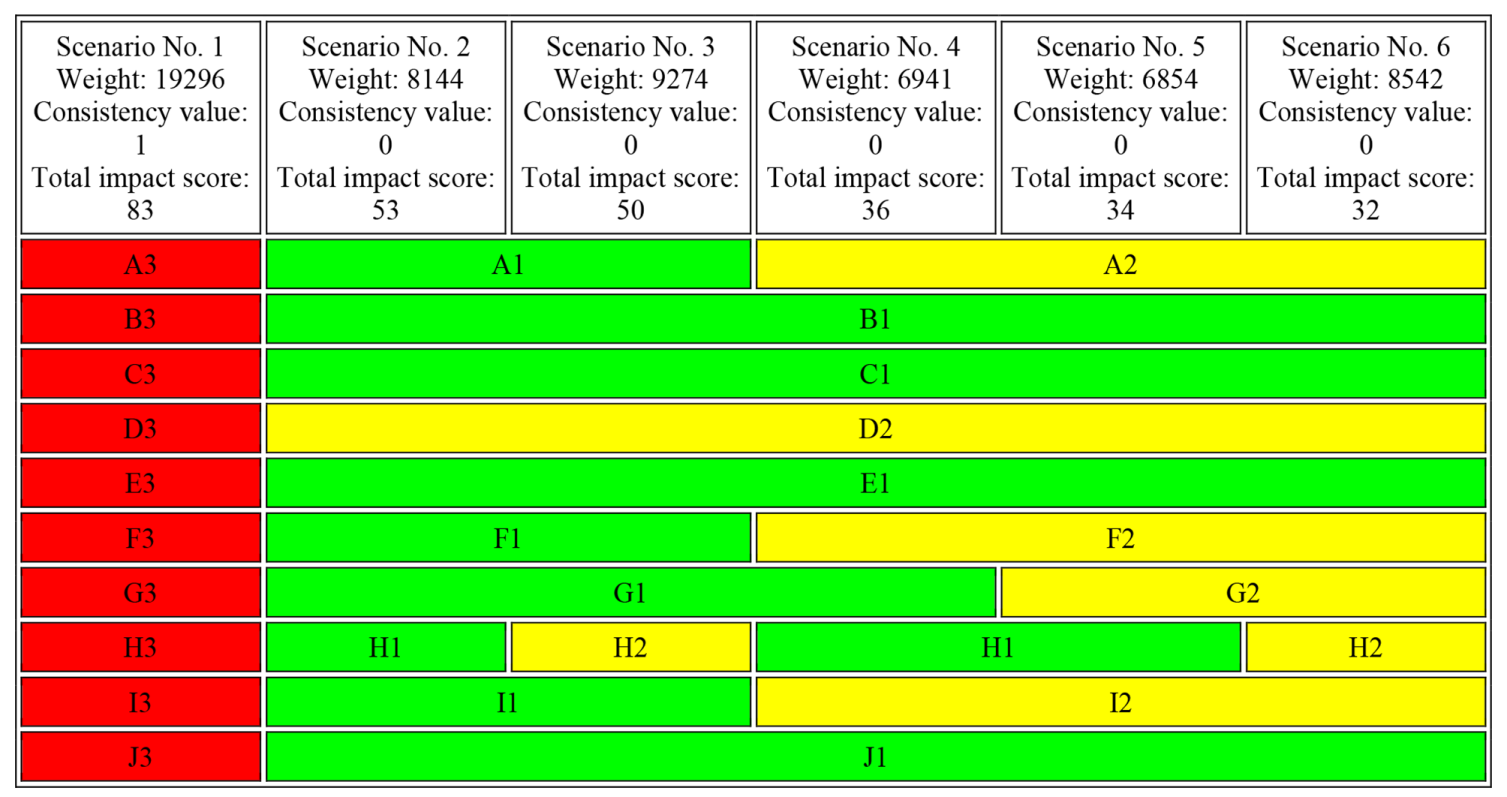
Scenarios with strong consistencies in the future of CHE in Iran

In Table 3, the consistency value of every possible state is indicated. According to the results, reducing the budget of the health system (A3) and the family physician plan and referral system are not implemented in Iran. (I3) has the highest consistency value. In fact, it is vital to budget deficit in Iran’s health system and Non-implementation of the family doctor and referral system are the most influential factor that posed numerous challenges in health expenditures.

**Table 3:** Value consistencies of every possible state

Descriptors	Possible states	Value consistency
A.	A3	16
I.	I3	15
G.	G3	13
F.	F3	12
H.	H3	6
J.	J3	5
B.	B3	4
C.	C3	4
D.	D3	1
E.	E3	1

### Validation studies

Identified information needs, personalities, and relevant usage scenarios were confirmed through member checks with study participants [27]. Information needs and scenarios of use were validated through a survey sent to all participants. All five participants completed the survey. Response to all survey items was 100% for all five participants. Participants were asked to assess each information need and the scenarios of use by answering the following questions:

- Is this information need accurately reflected?
- Does this scenario accurately describe activities that meet this need?

Participants were also asked to provide comments about each information need and scenario of use if they felt descriptions were not accurate or if additional feedback might be helpful.

### The scenario’s storyline

Once the key variables affecting households exposed to catastrophic and impoverishing health expenditures in Iran were identified, six possible scenarios were developed based on these variables. The scenarios were designed to help healthcare planners and policymakers in Iran anticipate and prepare for potential changes in the health system that could impact households’ financial protection.

#### Scenario 1: “Catastrophic cost crisis”

The worst-case scenario, paints a bleak picture of the future of catastrophic and impoverishing health expenditures in Iran. The budget of the health system is reduced (A3), which exacerbates the challenges already faced by the healthcare sector. Furthermore, the impact of sanctions is more severe (B3), which further limits the resources available for health care. In this scenario, informal payments are deeply ingrained in the healthcare sector (C3) and there is a severe conflict of interest (D3), leading to corruption and inefficiencies in the system. The increase in consumption of expensive high-tech health care services (E3) due to increased access to advanced medical technologies and willingness to pay, along with the inflation rate in the health sector increasing at a rate twice higher than the prevailing inflation rates (F3), leads to a significant increase in health care costs. The increase in consumer surplus due to induced demand (G3) further exacerbates the situation. Lack of access to up-to-date and integrated information systems (H3) limits the ability of healthcare providers to make informed decisions. In this scenario, the family physician plan and referral system are not implemented in Iran (I3), which limits access to primary care services and leads to an overreliance on costly secondary and tertiary care. Finally, the elimination of preferential currency subsidies and the sudden increase in medicines and medical device prices (J3) adds to the burden of healthcare costs and makes it more challenging for households to afford essential healthcare services. Overall, this scenario paints a grim picture of the future of health care in Iran, with households facing increasing financial hardships due to catastrophic health expenditures. Based on the story of the scenario, a suitable name for Scenario 1 could be “Catastrophic Cost Crisis”.

#### In scenario 2: “Sanction relief”

The optimistic scenario, envisions a future where economic sanctions on Iran are lifted (B1) and the budget of the healthcare system is increased (A1). Informal payments in the healthcare sector have been eradicated (C1), and there is only a moderate conflict of interest (D2), leading to a more efficient and effective healthcare system. Due to decreased access to advanced medical technologies and decreased willingness to pay, there is a decrease in the consumption of expensive high-tech healthcare services (E1). The inflation rate of the health system is reduced (F1), which makes healthcare services more affordable and accessible. There is a decrease in induced demand (G1), which helps manage healthcare costs. Integrated health information systems are developed in the health sector (H1), which helps improve healthcare quality and efficiency. The full and successful implementation of the family physician program (FPP) and referral system in Iran (I1) results in improved primary healthcare services. However, preferential currency subsidies are not eliminated, and expensive drugs are included in the basic health insurance benefit packages (J1), which makes essential healthcare services affordable to households. Overall, this scenario depicts a future in which the healthcare system in Iran is more sustainable, efficient, and accessible after the lifting of economic sanctions. Thus, the name “Sanction Relief” could be suitable for Scenario 2 as the story centers around the lifting of economic sanctions on Iran and its impact on the country’s healthcare sector.

#### Scenario 3: “Selective information access”

The realistic scenario, presents a mixed picture of the future of healthcare in Iran. On the one hand, the budget of the health system has been increased (A1), which allows for greater investment in the sector. The lifting of economic sanctions on Iran (B1) has also helped to alleviate some of the resource constraints that the healthcare sector was facing. Additionally, informal payments have been eradicated (C1), which has improved the efficiency of the system and reduced corruption. However, there is still a moderate conflict of interest in the sector (D2), which limits the effectiveness of the system. The decreased consumption of expensive high-tech health care services (E1) due to decreased access to advanced medical technologies and willingness to pay is a double-edged sword. On the one hand, it helps to reduce healthcare costs. On the other hand, it limits access to some of the most advanced and effective treatments available. The inflation rate of the health system is reduced (F1), which helps to control healthcare costs. The decrease in induced demand (G1) also contributes to this. Access to integrated and validated information for specific authorities (H2) helps healthcare providers to make informed decisions and improve the quality of care. The complete and successful implementation and performance of the FPP and referral system in Iran (I1) ensure access to primary care services and reduces reliance on costly secondary and tertiary care. Finally, it has been decided not to eliminate preferential currency subsidies, and expensive drugs have been included in the basic health insurance benefit packages (J1). While this decision has made healthcare more affordable, it has also led to increased healthcare costs in the long term. Overall, Scenario 3 presents a mixed picture of the future of healthcare in Iran. While there have been improvements in some areas, there are still significant challenges that need to be addressed to ensure that all Iranians have access to affordable, high-quality healthcare. The name “Selective Information Access” is appropriate for this scenario, as access to validated information is highlighted as a key factor in improving healthcare outcomes.

#### Scenario 4: “Technological ambiguity”

The believable scenario, envisions a future where the budget of Iran’s health system remains stable (A2) and economic sanctions are lifted (B1), providing some relief to the healthcare sector. In this scenario, informal payments are eradicated (C1) and there is only a moderate conflict of interest (D2), which reduces corruption and inefficiencies in the healthcare system. The consumption of expensive high-tech healthcare services decreases (E1) due to reduced access to advanced medical technologies and a decrease in willingness to pay for such services. The inflation rate in the healthcare sector remains constant (F2), which stabilizes healthcare costs. There is a decrease in induced demand (G1), which also helps to control healthcare costs. The development of integrated health information systems (H1) allows for more informed decision-making by healthcare providers. The family physician plan and referral system are implemented step-by-step in provinces (I2), increasing access to primary care services and reducing the reliance on costly secondary and tertiary care. Finally, preferential currency subsidies are not eliminated, and expensive drugs are included in basic health insurance benefit packages (J1), making it easier for households to afford essential healthcare services. Despite these improvements, the uncertainty around the adoption and use of new medical technologies creates a sense of “Technological Ambiguity” in the healthcare system, which may lead to inefficiencies and challenges in healthcare delivery.

#### Scenario 5: “Induced demand management”

The best-case scenario, depicts a future in which the budget of the healthcare system remains stable (A2), and economic sanctions on Iran are lifted (B1). Efforts to eradicate informal payments in the healthcare sector have been successful (C1), and there is only a moderate conflict of interest (D2), leading to a more efficient and effective healthcare system. Due to decreased access to advanced medical technologies and decreased willingness to pay, there is a decrease in the consumption of expensive high-tech healthcare services (E1). The inflation rate of the health system remains constant (F2), which ensures the affordability and accessibility of healthcare services. Moreover, there is no substantial change in induced demand (G2), which further helps to manage healthcare costs. Efforts have been made to develop integrated health information systems in the health sector (H1), which help healthcare providers make informed decisions. The family physician plan and referral system are implemented step-by-step in provinces (I2), which ensures that patients receive the appropriate level of care at the right time and reduces healthcare costs. The preferential currency subsidies are not eliminated, and expensive drugs are included in the basic health insurance benefit packages (J1), which makes essential healthcare services affordable to households. Overall, this scenario provides a hopeful future for the healthcare system in Iran, where healthcare services are accessible, affordable, and of high quality. The name “Induced Demand Management” is suitable for scenario 5 because it highlights the effective management of induced demand in the healthcare sector, which plays a crucial role in reducing healthcare costs and ensuring that healthcare services are accessible and affordable to households.

#### Scenario 6: “Incremental reforms”

The high desirable scenario, describes a future in which the budget of the healthcare system remains stable (A2), and economic sanctions on Iran are lifted (B1). Informal payments in the healthcare sector have been eradicated (C1), and there is only a moderate conflict of interest (D2), leading to a more efficient and effective healthcare system. Due to decreased access to advanced medical technologies and decreased willingness to pay, there is a decrease in the consumption of expensive high-tech healthcare services (E1). The inflation rate of the health system remains constant (F2), which ensures the affordability and accessibility of healthcare services. There is no substantial change in induced demand (G2), which helps manage healthcare costs. Specific authorities have access to integrated and validated information (H2), which helps them make informed decisions. A step-by-step approach is taken to eliminate preferential currency subsidies and increase the price of medicines and medical devices (I2), which gradually reduces the burden on the healthcare system. However, preferential currency subsidies are not eliminated, and expensive drugs are included in the basic health insurance benefit packages (J1), which makes essential healthcare services affordable to households. Overall, this scenario depicts a future in which the healthcare system in Iran undergoes incremental reforms, leading to a more sustainable and efficient healthcare system. The most suitable name for scenario 6 is “Incremental Reforms”, as it portrays a future in which the healthcare system in Iran is undergoing gradual improvements to become more efficient, accessible, and sustainable. The step-by-step approach taken to eliminate preferential currency subsidies and increase the price of medicines and medical devices is a clear indication of the incremental reforms being implemented.

Scenario 1"Catastrophic Cost Crisis”, which predicts catastrophic health expenditures, reduced health budgets, severe economic sanctions, corruption, and an increase in expensive high-tech healthcare services, was found to be the most believable scenario. Scenarios 2 and 3 have stable characteristics and are not appropriate for designing a strategy. Scenarios 4, 5, and 6 have idealistic for the future of the health system in Iran.

## Discussion

In developing countries such as Iran, the reliance on out-of-pocket (OOP) expenditures for healthcare financing has resulted in a concerning rise in households exposed to catastrophic and impoverishing health expenditures. This research aimed to evaluate the future impacts of various drivers, macro trends, and associated uncertainties shaping catastrophic and impoverishing health expenditures in Iran. Alternative scenarios for the year 2030 were presented through a qualitative methodology that employed scenario planning techniques, cross-impact analysis matrix tools, and the MicMac software to identify and analyze the drivers of uncertainty surrounding these health expenditures.

The Cross-Impact Analysis (CIA) technique, as a means of futures research, provided valuable insights by revealing the characteristic role and importance of variables in relation to each other in the system. This method allowed us to consider the potential impacts that future events may have on each other. Subsequently, the Scenario Wizard software, in collaboration with an expert panel, was utilized to pinpoint key uncertainties and articulate potential scenarios for the evolution of catastrophic and impoverishing health expenditures by 2030. The integration of innovative futures study methods, particularly scenario building, has become increasingly beneficial in contemporary healthcare planning and management, offering the flexibility needed to formulate strategic solutions for nationwide health economic issues.

The study further employed Cross-Impact Balance Analysis (CIB) as futures study tools, identifying the roles and significance of each variable within Iran’s health system. A systematic framework, based on CIA, established contextual relationships among the 29 variables affecting households exposed to catastrophic and impoverishing health expenditures in Iran. Fuzzy MICMAC analysis was then applied to evaluate the interactions among the identified variables, leading to the identification of 10 key variables crucial for developing scenarios using the cross-impact algorithm:

- Var36 Budget deficit of the health system.
- Var53 Economic sanctions against Iran.
- Var25 Informal payments or under-the-counter payment.
- Var45 Conflict of interests of Iran’s health system decision-makers.
- Var21 Increasing consumption of expensive high-tech health care services.
- Var54 Inflation rate in the health sector.
- Var33 Induced demand (consumer or supplier).
- Var44 Lack of reliable and transparent electronic information systems.
- Var52 Lack of implementation of family physician and referral system in the whole country.
- Var41 The increase in the price of medicine and medical devices due to the increase in the exchange rate and the removal of the preferential currency subsidy.

According to cross-impact balance analysis, six scenarios with strong consistencies were identified, exploring the future of healthcare expenditure in Iran under different economic and policy environments. Scenario 1 emerged as the most plausible future, portraying a grim picture of healthcare in Iran characterized by reduced funding, severe economic sanctions, and widespread informal payments and corruption, resulting in escalating healthcare costs and financial hardships for households. In Scenario 1, the variable A3 (reducing the budget of the health system) exhibited the highest consistency value. The primary reason for the substantial out-of-pocket expenses and increasing catastrophic and impoverishing health expenditures was the constrained budget of the health sector compared to the national budget, leading to a general government budget deficit. Addressing this issue requires moving toward operational budgeting, cost-efficiency management (evidence-based decision-making), and creating a system for the timely receipt of revenues.

I3 (the Family Physician Program and referral system not implemented in Iran) was the second variable with high consistency in Scenario 1. Successful implementation of family physician policies depends on the political, economic, social, and cultural context in the country, emphasizing the need to consider these factors for effective policy execution.

G3 (the increase in consumer surplus due to induced demand) emerged as the third variable with high consistency in the first scenario. Policies to impede physician-induced demand, such as implementing the family physician plan at the national level, developing clinical guidelines for family physicians, and establishing a comprehensive health services system, are crucial to addressing this issue.

F3 (the inflation rate in the health sector increases at a rate twice higher than prevailing inflation rates) was the fourth variable with high consistency in Scenario 1. Effective control of the health inflation rate can be achieved through increasing insurance coverage, which could decrease the health inflation rate by controlling prices and compelling health service providers to stabilize prices.

H3 (lack of access to up-to-date and integrated information systems) emerged as the fifth variable with high consistency in Scenario 1. Establishing a comprehensive up-to-date information system is crucial for improving governance, surveillance, responsibility, resource mobilization, and financing functions in the health sector. This involves substituting traditional health service delivery structures with health information technologies, creating a national online data warehouse for health insurance enrollees, and strengthening the financing function.

J3 (eliminate preferential currency subsidies and sudden increase in medicines and medical device prices) was the sixth variable with high consistency in Scenario 1. The elimination of foreign currency subsidies for medicines would adversely affect accessibility, and careful consideration of political context and the addressing of exchange rate gaps are necessary.

B3 (sanctions have more severe consequences) emerged as the seventh variable with high consistency in Scenario 1. Intensifying sanctions led to an increased inflation rate in the health sector, decreased services by insurance companies, and a subsequent rise in out-of-pocket payments, negatively affecting access to healthcare.

C3 (informal payments are deeply ingrained in the healthcare sector) was the eighth variable with high consistency in Scenario 1. Mitigating the persistence of informal payments and high co-payments requires realistic tariff valuation, increased monitoring, timely payment to healthcare providers, deterrence laws, increased supervision and coordination between relevant organizations, ethics training, and performance-based payment.

D3 (severe conflict of interest) was the ninth variable with high consistency in Scenario 1. The complexity and conflict of interest in the health system require the adoption of conflict-of-interest policies and procedures, including annual conflict disclosures, to effectively manage this phenomenon.

E3 (increasing consumption of expensive high-tech health care services due to increased access to advanced medical technologies and an increase in willingness to pay) was the tenth variable with high consistency in Scenario 1. Health Technology Assessment is crucial in ensuring the maximum health benefit for the community and preventing the emergence of inefficient technologies.

The results underscore that Scenario 1, driven by a reduction in the health system budget, is a significant driver scenario in Iran’s health system, exacerbating households’ exposure to catastrophic and impoverishing health expenditures. It paints a challenging picture of the future of healthcare in Iran, with decreased funding, severe economic sanctions, and deeply ingrained informal payments and corruption leading to skyrocketing healthcare costs and financial hardships for households.

In this scenario, addressing the insufficient budget of the health sector, the successful implementation of family physician policies, policies to impede physician-induced demand, controlling the health inflation rate, establishing comprehensive up-to-date information systems, addressing exchange rate gaps, mitigating informal payments, managing conflicts of interest, and adopting health technology assessment are critical steps for improvement and strengthening of the health system. This study offers valuable insights into potential future scenarios of catastrophic and impoverishing health expenditures in Iran. By understanding and addressing the key variables, policymakers can make informed decisions to mitigate the impact of future challenges, ensuring a more resilient and equitable healthcare system for the nation.

While cross-impact analysis offers valuable insights, it is essential to acknowledge the inherent limitations associated with its application. The methodology relies on assumptions and expert opinions, introducing a degree of subjectivity into the analysis. The scenarios presented in this study are exploratory and based on the current understanding, and it is crucial to recognize that future developments may influence the actual trajectory of catastrophic and impoverishing health expenditures in Iran. The uncertainties inherent in projecting future events highlight the need for caution in interpreting the results and the importance of regularly revisiting and updating scenarios to reflect evolving realities.

Scenario analysis, encompassing methodologies such as MICMAC and Scenario Wizard analyses, is widely regarded as a more effective method compared to many contemporary approaches. However, it is imperative to highlight certain limitations associated with these techniques. A key consideration is the substantial reliance on the knowledge and expertise of the expert panel, and the presence of biases among panel members can significantly impact the results. To address this challenge, assembling a multidisciplinary team is essential, ensuring a diversity of perspectives and expertise that contributes to a more comprehensive and unbiased assessment.

The process of developing scenarios for future studies represents a simplified approach when contrasted with the intricate complexities and contradictions inherent in the real world. It is essential for readers to recognize that scenarios, by their nature, involve a level of abstraction from reality. Despite these challenges, the identification of critical factors, uncertainties, and potential scenarios equips decision-makers with the necessary context to navigate intricate interactions and emerging changes within the healthcare system. This facilitates the establishment of a foundation for prioritizing and implementing effective strategies in response to plausible future scenarios.

Despite the acknowledged limitations, the study’s robustness is grounded in its comprehensive analysis and identification of key variables influencing future health expenditures in Iran. The rigorous design of the methodology, including the use of cross-impact analysis tools such as CIA and CIB, adds to the strength of the study. While subjectivity is inherent in expert-driven methodologies, the large set of variables considered, statistical analyses employed, and scenario-building approach contribute to the overall reliability of the findings.

## Conclusion

This study underscores the importance of developing flexible strategies and scenarios for improving the health system in developing countries, such as Iran, and highlights the need to address key variables affecting households exposed to catastrophic and impoverishing health expenditures. Based on the findings of this study, key components were identified, and by structural analysis of the relationships between them in MicMac software, the variables “budget deficit”, “economic sanctions”, “informal payments”, “inflation rate”, “conflict of interests”, “induced demand”, “increasing consumption of expensive high-tech healthcare services”, “lack of reliable and transparent electronic information systems”, “lack of implementation of family physician and referral system”, and “increase in the price of medicine and medical devices” as drivers. By addressing these variables, policymakers can take steps to improve access to healthcare and protect households from financial hardship.

The six main scenario spaces were mapped and narrated, extracted from possible and compatible alternative futures based on the results of Scenario wizard software, which is a reasoned and reliable basis for designing any strategy and policy in the future of healthcare expenditure in Iran. These scenarios provide a helpful tool for Iranian healthcare planners and policymakers to identify potential challenges and develop strategies to address them.

The study recommends several policy options for Iran’s health system according to the driver scenario. These include implementing operational budgeting, cost-efficiency management, and timely receipt of revenues, as well as reinforcing governance, regulation, financing, payment, and behavior dimensions. Other policies include increasing insurance coverage, investing more in the health system, using health information technologies, and adopting a conflict-of-interest policy. Iran should also use Health Technology Assessment and implement universal healthcare to ensure maximum health and prevent financial hardship.

### Electronic supplementary material

Below is the link to the electronic supplementary material.

Supplementary Material 1

## Data Availability

All data generated or analyzed during this study were included in the published article.

## Abbreviations

LMICs: Low and middle-income countries
CHE: Catastrophic health expenditure
UHC: Universal health coverage
SDGs: Sustainable Development Goals
WHO: World Health Organization
OOPs: Out-of-pocket payments
EMRO: Eastern Mediterranean Regional Office
STEEP: Social, technical, economic, environmental, and political
MOHME: Ministry of Health and Medical Education
CIA: Cross‑impact analysis
CIB: Cross-impact balance analysis
MDI: Matrix of direct influence
