# Supplementary material for: Exploring the driving forces and scenario analysis for catastrophic and impoverishing health expenditures in Iran
Source: BMC Health Serv Res. 2024 Feb 26;24:245. doi: 10.1186/s12913-024-10551-w (PMC10898180; doi:10.1186/s12913-024-10551-w)
Supplement: Supplementary file 1 — Supplementary Material 1 [file 12913_2024_10551_MOESM1_ESM.docx]

## **Supplementary Information**

Supplementary 1. Design and consecutive phases of the study; Supplementary 2. Overview of the approach and method; Supplementary 3. Search strategy for the main components; Supplementary 4. PRISMA-SCR flow diagram; Supplementary 5. Interview Guide; Supplementary 6. Hypothetical sample of spatial structure of variables; Supplementary 7. Summary Characteristics of Included Studies; Supplementary 8. Factors associated with catastrophic health expenditure; Supplementary 9. Factors associated with catastrophic health expenditure; Supplementary 10. Average score of key variables; Supplementary 11. Wilson Matrix used to prioritize scenario driver; Supplementary 12. Completed Cross Impact Matrix of key factors; Supplementary 13. Potential Direct Influences Matrix; Supplementary 14. The Matrix of Direct Effects of Factors; Supplementary 15. he Matrix of indirect Effects of Factors; Supplementary 16. Direct influence and dependence of variables.

### **Supplementary 1. Design and consecutive phases of the study**

### **Supplementary 2. Overview of the approach and method**

Scoping review

&

Semi-Structured interview

Determination of key factors and trends

Designing cross impact matrix

MICMAC Analyze

Designing Cross Impact Balance Matrix

Scenario Wizard

Compatible Scenarios

Determination of driving Force

Determination of variable

Scenario’s Storyline

Practical strategies

Expert panel

### **Supplementary 3. Search strategy for the main components**

| Searched databases: PubMed, Scopus, Science Direct, Web of Science, and Scientific Information Database | |
| --- | --- |
| Strategy #1 AND #2 AND #3 AND #4 AND #5 | |
| #1 | Direct Expenditure **OR** Health expenditures **OR** Out of Pocket Expenditures **OR** Out-of-Pocket Costs **OR** Out-of-Pocket Payments **OR** Out-of-Pocket Spending **OR** Direct expenditure **OR** Indirect expenditure **OR** Health Care Cost **OR** Medical Care Costs **OR** Treatment Costs **OR** catastrophic health expenditure **OR** Health impoverishment **OR** impoverishing health expenditures **OR** impoverishment due to health costs **OR** Health poverty. |
| #2 | Health System Equity **OR** value-based health insurance **OR** Cost sharing **OR** Insurance Design **OR** Fair financial contribution **OR** socioeconomic inequality **OR** Financial inequity **OR** Disparity |
| #3 | Factors **OR** characteristics **OR** Driving forces **OR** Characteristic **OR** Criteria **OR** Determinants |
| #4 | Patient **OR** Family **OR** Household |
| #5 | Iran |
| Limitations Language: articles with at least an abstract in English. | |

### **Supplementary 4. PRISMA-SCR flow diagram**

[Identification](http://prisma-statement.org/prismastatement/flowdiagram.aspx)

[Studies extracted from the six above-mentioned databases](http://prisma-statement.org/prismastatement/flowdiagram.aspx)

[(n=848)](http://prisma-statement.org/prismastatement/flowdiagram.aspx)

[Screening](http://prisma-statement.org/prismastatement/flowdiagram.aspx)

[Records screened for titles-abstract-keywords-conclusions
(n = 230)](http://prisma-statement.org/prismastatement/flowdiagram.aspx)

[Duplicates removed
(n = 81)](http://prisma-statement.org/prismastatement/flowdiagram.aspx)

[Full-text articles assessed for eligibility
(n =149 )](http://prisma-statement.org/prismastatement/flowdiagram.aspx)

[Records excluded](http://prisma-statement.org/prismastatement/flowdiagram.aspx)

[(n = 38)](http://prisma-statement.org/prismastatement/flowdiagram.aspx)

[Eligibility](http://prisma-statement.org/prismastatement/flowdiagram.aspx)

[Records identified through PubMed database](http://prisma-statement.org/prismastatement/flowdiagram.aspx)

[(n=481)](http://prisma-statement.org/prismastatement/flowdiagram.aspx)

[Additional records identified through other sources](http://prisma-statement.org/prismastatement/flowdiagram.aspx)

[(n= 0)](http://prisma-statement.org/prismastatement/flowdiagram.aspx)

[Included](http://prisma-statement.org/prismastatement/flowdiagram.aspx)

[Final report extracted from WHO, World bank, and google scholar](http://prisma-statement.org/prismastatement/flowdiagram.aspx)

[(n =1)](http://prisma-statement.org/prismastatement/flowdiagram.aspx)

[Studies included in qualitative synthesis](http://prisma-statement.org/prismastatement/flowdiagram.aspx)

[(n =112)](http://prisma-statement.org/prismastatement/flowdiagram.aspx)

[Records identified through Scopus database](http://prisma-statement.org/prismastatement/flowdiagram.aspx)

[(n=47)](http://prisma-statement.org/prismastatement/flowdiagram.aspx)

[Records identified through Web of science databases](http://prisma-statement.org/prismastatement/flowdiagram.aspx)

[(n= 112)](http://prisma-statement.org/prismastatement/flowdiagram.aspx)

[Records identified through ProQuest database](http://prisma-statement.org/prismastatement/flowdiagram.aspx)

[(n=173)](http://prisma-statement.org/prismastatement/flowdiagram.aspx)

[Records identified through Persian databases](http://prisma-statement.org/prismastatement/flowdiagram.aspx)

[(n= 35)](http://prisma-statement.org/prismastatement/flowdiagram.aspx)

[Records deleted after studying title (n = 500)](http://prisma-statement.org/prismastatement/flowdiagram.aspx)

[Records excluded](http://prisma-statement.org/prismastatement/flowdiagram.aspx)

[(n=81)](http://prisma-statement.org/prismastatement/flowdiagram.aspx)

- [Non relative](http://prisma-statement.org/prismastatement/flowdiagram.aspx)

### **Supplementary 5. Scoping Reviews (PRISMA-ScR) Checklist**

| **SECTION** | **ITEM** | **PRISMA-ScR CHECKLIST ITEM** | **REPORTED ON PAGE #** |  |
| --- | --- | --- | --- | --- |
| **TITLE** | | | | |
| Title | 1 | Identify the report as a scoping review. | Scoping Review on Determinants of Catastrophic and Impoverishing Healthcare Expenditures in Iran |  |
| **ABSTRACT** | | | | |
| Structured summary | 2 | Provide a structured summary that includes (as applicable): background, objectives, eligibility criteria, sources of evidence, charting methods, results, and conclusions that relate to the review questions and objectives. | Background:  The high reliance on out-of-pocket (OOP) payments for health financing in Iran has resulted in various inequity problems, notably catastrophic health expenditure (CHE) and impoverishment. This scoping review aims to comprehend the variations in CHE and impoverishment over the past 20 years, identify the underlying determinants of CHE, and explore the associated inequalities.  Objectives:  Understand variations in catastrophic health expenditure (CHE) and impoverishment in Iran.  Identify the key determinants influencing the rate of CHE.  Assess the inequality associated with CHE.  Eligibility Criteria:  Studies included in the review reported the rate of CHE, impoverishment, inequality, and influencing factors. The review focused on data from January 1, 2000, to December 30, 2021.  Sources of Evidence:  Systematic searches were conducted on PubMed, Scopus, Web of Science, ProQuest, Scientific Information Database, IranMedex, IranDoc, Magiran Science, Google Scholar, and grey literature.  Charting Methods:  The scoping review followed Arksey and O’Malley’s framework, employing systematic searches and including studies that met the specified criteria. Data were charted using simple descriptive statistics and narrative synthesis.  Results:  From the 110 included articles, the average incidence of CHE was found to be 3.19% at the 40% threshold. Approximately 3.21% of households experienced impoverishment. Key drivers influencing CHE included household economic status, place of residence, health insurance status, household size, head of the household’s gender, education level, employment status, presence of household members under 5/ above 60 years old, chronic diseases (especially cancer and dialysis), disability, utilization of inpatient and outpatient services, dentistry services, medicines and equipment, and low insurance coverage.  Conclusions:  The scoping review calls for intensified health policies and financing structures in Iran to ensure more equitable access, particularly for the poorest and most vulnerable populations. Additionally, the government is urged to adopt effective measures in inpatient and outpatient care, dental services, medicines, and equipment to address the identified determinants of catastrophic health expenditure and impoverishment. |  |
| **INTRODUCTION** | | | | |
| Rationale | 3 | Describe the rationale for the review in the context of what is already known. Explain why the review questions/objectives lend themselves to a scoping review approach. | Variations in Distribution: The scoping review aims to explore variations in the distribution of CHE and impoverishment. Taking a broad approach, it allows for the identification of diverse factors contributing to these outcomes and an understanding of how they are distributed among different population groups.  Associated Risk Factors: Understanding the associated risk factors is crucial for effective policy design. A scoping review is suitable for capturing a wide range of determinants, going beyond the scope of traditional systematic reviews to provide a holistic view of the factors influencing CHE and impoverishment.  Addressing Evidence Gap: By focusing on population-based studies and aiming to fill the existing evidence gap, the scoping review intends to offer a more complete and nuanced understanding of the CHE and impoverishment landscape in Iran.  Flexibility in Study Designs: The scoping review approach allows for the inclusion of diverse study designs, acknowledging the varied methodologies employed in existing literature. This flexibility is essential for capturing the richness and complexity of available evidence.  In summary, the scoping review is designed to address the limitations in the current literature by adopting a broad and inclusive approach. It aims to provide policymakers in Iran with a comprehensive overview of the variations, risk factors, and trends associated with CHE and impoverishment, ultimately supporting the development of targeted and evidence-based health policies. |  |
| Objectives | 4 | Provide an explicit statement of the questions and objectives being addressed with reference to their key elements (e.g., population or participants, concepts, and context) or other relevant key elements used to conceptualize the review questions and/or objectives. | 1. Population/Participants:  Primary Incidence and Intensity of Catastrophic Healthcare Expenditures (CHE) and Impoverishment:  Question: What is the incidence and intensity of catastrophic healthcare expenditures and impoverishment in the Iranian population?  Objective:  -To identify and describe studies reporting on the primary incidence and intensity of catastrophic healthcare expenditures and impoverishment in Iran.  -To determinants of Catastrophic Healthcare Expenditures (CHE) and Impoverishment:  Question: What are the determinants influencing catastrophic healthcare expenditures and impoverishment in Iran?  Objective: To explore and summarize studies investigating the determinants associated with catastrophic healthcare expenditures and impoverishment in the Iranian context.  2. Concepts:  Determinants of Catastrophic Healthcare Expenditures (CHE) and Impoverishment:  Question: What are the determinants influencing catastrophic healthcare expenditures and impoverishment in Iran?  Objective: To identify and categorize determinants, contributing to catastrophic healthcare expenditures and impoverishment.  3. Context:  According to the 2019 report by the WHO in the Eastern Mediterranean Regional Office (11), a huge portion of health services in Iran is paid OOPs, which is around 39.49% of the current health expenditure while this portion amongst EMRO countries is around 36.22%. |  |
| **METHODS** | | | | |
| Protocol and registration | 5 | Indicate whether a review protocol exists; state if and where it can be accessed (e.g., a Web address); and if available, provide registration information, including the registration number. | This scoping review did not have a review protocol. |  |
| Eligibility criteria | 6 | Specify characteristics of the sources of evidence used as eligibility criteria (e.g., years considered, language, and publication status), and provide a rationale. | Inclusion Criteria:  Source of Information:  Criteria: Studies published in peer-reviewed journals or publicly accessible grey literature.  Rationale: Ensures that the included studies have undergone a review process or are publicly available, enhancing the reliability and accessibility of the information.  Time Frame:  Criteria: Studies conducted from the year 2000 to December 2021.  Rationale: Focuses the review on the most recent two decades, providing a contemporary perspective on catastrophic healthcare expenditures and impoverishment in Iran.  Language:  Criteria: Abstracts in English; full-text in both English and Persian languages.  Rationale: The inclusion of English and Persian languages facilitates a comprehensive review of the available literature, considering the primary languages used in Iranian research.  Research Location:  Criteria: Studies conducted in Iran.  Rationale: Limits the review to studies directly relevant to the Iranian context, ensuring the applicability of findings to the target population.  Study Population:  Criteria: Studies focusing on all population groups, including vulnerable groups such as people with disabilities, diseases, the elderly, or children, in rural and urban areas in Iran.  Rationale: Encompasses a broad spectrum of the Iranian population, allowing for a comprehensive understanding of the determinants of catastrophic healthcare expenditures and impoverishment across diverse groups.  Types of Interventions:  Criteria: Factors or determinants influencing catastrophic healthcare expenditures and impoverishment of Iranian households, including the incidence of CHE and healthcare impoverishment. Also, the most important indicators of financial protection.  Rationale: This directly aligns with the aim of the scoping review, focusing on the key factors influencing catastrophic health expenditures and household impoverishment in Iran.  Type of Studies:  Criteria: Any primary study in English or Persian assessing, measuring, or reporting catastrophic healthcare expenditures and/or household health impoverishment due to out-of-pocket payments in healthcare. Also, studies assessing factors affecting them across demographics and diseases, were conducted in Iran. All study designs, including various observational and experimental designs, were considered for inclusion.  Rationale: Promotes inclusivity by considering a wide range of study designs, ensuring a comprehensive exploration of the subject matter.  Exclusion Criteria:  Type of Studies:  Criteria: Methodological studies, discussion papers, general literature reviews, qualitative studies, case reports, case series, systematic reviews, narrative reviews, letters to editors, commentary pieces, and study protocols.  Rationale: Excludes study types that may not directly contribute to the primary objectives of the scoping review, focusing on methodologically rigorous and relevant primary studies.  Language:  Criteria: Any language other than English or Persian.  Rationale: Limits the review to languages that align with the primary languages used in Iranian research, ensuring meaningful inclusion and analysis.  Time Restriction:  Criteria: Studies conducted before the year 2000.  Rationale: Focuses on recent literature to provide a contemporary overview of catastrophic healthcare expenditures and impoverishment in Iran, excluding potentially outdated information.  The eligibility criteria are carefully designed to ensure the relevance, quality, and inclusivity of studies included in the scoping review, aligning with the review's specific objectives and scope. |  |
| Information sources* | 7 | Describe all information sources in the search (e.g., databases with dates of coverage and contact with authors to identify additional sources), as well as the date the most recent search was executed. | Electronic Database Searches:  Databases: Web of Science (WoS), PubMed, Scopus Elsevier, Magiran, IranDoc, IranMedex, Scientific Information Database (SID), Google Scholar.  Dates of Coverage: From the year 2000 to December 2021.  Search Strategy: Developed by a medical research librarian, the search strategy included text words and Medical Subject Headings (MeSH) terms related to "out-of-pocket expenditure," "financial risk protection," "catastrophic health expenditure," and "impoverishment" in English and Persian. No search filters were used to generate a broad list of studies suitable for answering the research questions.  Search Execution: The search was executed on the 30th of December 2021.  Grey Literature Searches:  Grey Literature Sources: Virtual libraries of relevant organizations such as the World Health Organization (WHO), the World Bank (WB), and the Ministry of Health and Medical Education (MOHME).  Search Method: Followed the methods outlined in "Grey Matters: a practical tool for searching health-related grey literature" (20).  Objective: To include relevant grey literature sources in addition to peer-reviewed publications.  Expert Consultation:  Consultation Process: Expert consultation was conducted to identify additional sources or studies that might not be captured through the database and grey literature searches.  Objective: To ensure a comprehensive inclusion of relevant studies and information.  Three-Step Search Strategy:  Step 1: Limited search of two initial databases - Medline and PubMed. Analysis of subject headings and search terms based on titles and abstracts identified.  Step 2: Conducted a second search using all identified subject headings and keywords across all specified databases.  Step 3: Searched the reference lists of all articles selected for inclusion in the review for additional studies.  Objective: Employed a systematic and iterative approach to maximize the identification of relevant literature.  Communication with Authors:  Author Contact: If required, authors of relevant studies or reviews were approached for supplementary information.  Objective: To obtain additional data or clarification on relevant studies.  Record of Searches:  Documentation: An updated record of searches was kept to track when the same search terms were applied in other databases.  Objective: To maintain transparency and ensure the reproducibility of the search process. |  |
| Search | 8 | Present the full electronic search strategy for at least 1 database, including any limits used, such that it could be repeated. | The full electronic search strategy for PubMed: ((((((((((((((((((((((((((((((((((((((("health expenditures"[MeSH Terms])) OR ("health care costs"[MeSH Terms])) OR ("health care cost*"[Title/Abstract])) OR ("health cost*"[Title/Abstract])) OR ("treatment cost*"[Title/Abstract])) OR ("health expenditure*"[Title/Abstract])) OR ("direct expenditure*"[Title/Abstract])) OR ("out-of-pocket expenditure*"[Title/Abstract])) OR ("out-of-pocket cost*"[Title/Abstract])) OR ("out-of-pocket payment*"[Title/Abstract])) OR ("out-of-pocket spending*"[Title/Abstract])) OR ("indirect expenditure*"[Title/Abstract])) OR ("Health impoverishment"[Title/Abstract])) OR ("impoverishing health expenditures"[Title/Abstract])) OR ("private health financing"[Title/Abstract])) OR("catastrophic health expenditure"[Title/Abstract])) OR ("Health poverty"[Title/Abstract])) AND ("health equity"[MeSH Terms])) OR ("value-based health insurance"[MeSH Terms])) OR ("Insurance Design"[Title/Abstract])) OR ("Health System Equity"[Title/Abstract])) OR ("Fair financial contribution"[Title/Abstract])) OR ("socioeconomic inequality"[Title/Abstract])) OR (Disparity[Title/Abstract])) OR ("Financial inequity"[Title/Abstract])) OR ("cost sharing"[Title/Abstract])) AND ("socioeconomic factors"[MeSH Terms])) OR ("family characteristics"[MeSH Terms])) OR (Trend*[Title/Abstract])) OR (Determinants[Title/Abstract])) OR (Factors[Title/Abstract])) OR (characteristics[Title/Abstract])) OR ("Driving forces"[Title/Abstract])) AND ("Health Policy"[MeSH Terms])) OR ("Healthcare Policies"[Title/Abstract])) OR ("National Health Policies"[Title/Abstract])) OR (strategies[Title/Abstract])) AND (patients[MeSH Terms])) OR (Household*[Title/Abstract])) AND (Iran[MeSH Terms]) |  |
| Selection of sources of evidence† | 9 | State the process for selecting sources of evidence (i.e., screening and eligibility) included in the scoping review. | The process for selecting sources of evidence in the scoping review involved several steps, guided by the Population, Concept, Context (PCC) framework recommended by the Joanna Briggs Institute (JBI). Here is an overview of the selection process:  Eligibility Criteria Establishment:  The eligibility criteria were established based on the PCC framework. These criteria included aspects such as the source of information, time frame, language, research location, study population, types of interventions, and types of studies and articles.  Exploratory Search and Discussions:  An initial exploratory search was conducted to identify relevant concepts and discuss the scope of the review among the review team members. This step helped in refining the eligibility criteria and focusing on key elements.  The eligibility criteria were documented, specifying both inclusion and exclusion criteria. This table provided a clear reference for screening studies during the review process.  Search Strategy Execution:  Electronic database searches were conducted using a comprehensive search strategy covering multiple databases (Web of Science, PubMed, Scopus, Elsevier, Magiran, IranDoc, IranMedex, Scientific Information Database, and Google Scholar) from the year 2000 to December 2021.  Gray Literature Search:  Gray literature searches were performed in virtual libraries of relevant organizations such as the World Health Organization (WHO), World Bank (WB), and Ministry of Health and Medical Education (MOHME).  Study Selection (Two-Step Process):  After removing duplicates, the first step involved screening the title, abstract, keywords, and conclusions of each article by one reviewer (M. H). This step aimed to exclude studies not meet the inclusion criteria or were unrelated to the research questions.  In the second step, the remaining studies were divided among two reviewers (M. H, I. MA) for a more detailed review. Each article was independently screened for inclusion, exclusion, or uncertainty. Disagreements were resolved through consultation with the rest of the reviewers.  Data Charting:  Data charting (extraction) was performed using an Excel worksheet and a data extraction form developed by the authors. Two reviewers (M. H, S. G.) independently extracted data from selected studies.  Synthesis of Results:  The analytical framework was used for data synthesis and thematic analysis. The main reviewer (M. H, I. MA, MR. M) extracted and analyzed data from all articles in consultation with other authors. Descriptive statistics and narrative synthesis were used due to the diversity of study designs.  This process ensured a systematic and transparent approach to selecting sources of evidence for the scoping review, aligning with the established eligibility criteria and the overarching aim of the study. |  |
| Data charting process‡ | 10 | Describe the methods of charting data from the included sources of evidence (e.g., calibrated forms or forms that have been tested by the team before their use, and whether data charting was done independently or in duplicate) and any processes for obtaining and confirming data from investigators. | The methods of charting data from the included sources of evidence in the scoping review involved a systematic and collaborative approach. Here are the key aspects of the data charting process:  Charting Form Development:  A charting form was developed by the authors to record study characteristics and variables relevant to the review question. The form aimed to capture information related to the incidence and intensity of catastrophic healthcare expenditures (CHE) and impoverishment, as well as the determinants of CHE in the Iranian context.  Calibration and Testing:  The charting form was calibrated and tested by two reviewers (MH, SG) to ensure its effectiveness in extracting relevant data. This calibration process likely involved a pilot test on a small sample of included studies to refine the form and address any discrepancies or challenges in data extraction.  Data Extraction:  Two reviewers (MH, SG) independently conducted data extraction from the selected studies using the charting form. The form covered a range of information, including study characteristics, key results, and variables related to CHE and impoverishment.  Collaborative Data Charting:  The data charting process was a collaborative effort, with two reviewers working independently on extracting data. This approach helps ensure the reliability and accuracy of the extracted information. Any disagreements or uncertainties in data extraction were likely resolved through discussion and consensus among the reviewers.  Iterative Process:  The extraction process was iterative, meaning that the charting form was continuously updated and refined as the scoping review progressed. This flexibility allowed the team to adapt the charting form based on the evolving needs of the review and the nature of the included studies.  Contacting Authors for Supplementary Information:  Authors of studies included in the review were contacted to obtain or confirm information. This step aimed to enhance the completeness and accuracy of the extracted data, especially when certain details were not fully reported in the published studies.  Reviewer Collaboration:  Collaboration among the reviewers (MH, SG, and potentially others involved) was crucial throughout the charting process. Regular communication and discussion helped ensure consistency in data extraction and adherence to the scoping review objectives.  Overall, the data charting methods employed a systematic and collaborative approach, with a focus on transparency, reliability, and flexibility to accommodate the diverse nature of the included studies. |  |
| Data items | 11 | List and define all variables for which data were sought and any assumptions and simplifications made. | The scoping review sought data on a variety of variables related to catastrophic healthcare expenditures (CHE) and impoverishment in Iran. The identified variables were categorized into different groups, and they encompassed aspects related to household characteristics, healthcare utilization, health expenditure indicators, and macroeconomic indicators. Below is a list of the variables for which data were sought, along with brief definitions:  Socioeconomics Characteristics of Household:  Household Economic Status (Q1 vs. Q5): Classification of households based on economic status, comparing the first (Q1) and fifth (Q5) quintiles.  Place of Residence: Categorization of households by residence type, such as urban, rural, or remote areas.  Health Insurance Status of Household: The presence or absence of health insurance coverage for the household.  Supplementary Insurance Status of Household: The status of supplementary insurance coverage for both the head and members of the household.  Wealth Index (Income Deciles): Division of households into income deciles based on their wealth index.  Homeownership: Whether the household owns its residence.  Type of Health Insurance: The specific type of health insurance coverage held by the household.  Per Capita Household or Housing Infrastructure: Average household expenditure per person or per housing unit.  Demographic Characteristics of Household:  Household Size: The number of individuals living in a household.  Gender of Household Head (HHH): The gender of the head of the household.  Education Level of HHH: The highest level of education attained by the head of the household.  Employment Status of HHH or Members: The employment status of the head of the household or household members.  Education Level of Household Members or Patient: The highest level of education attained by household members or the patient.  Age of HHH: The age of the head of the household.  Marital Status of HHH: The marital status of the head of the household.  Gender of Patients: The gender of individuals requiring healthcare in the household.  Male Ratio of Household: The proportion of males in the household.  Households in Which the Head Is a Student: Identification of households where the head is a student.  Age of Patient at Disease Incidence: The age of the patient when the disease occurred.  Vulnerable Persons in Household:  Having Elderly Member (Over 60 Years of Age) in Household: Identification of households with elderly members.  Have Under 5 Years Children in Household: Identification of households with children under 5 years old.  Having Member with Chronic Disease and NCDs: Identification of households with members having chronic diseases, including cancer, renal dialysis, MS, SMDs, and diabetes.  Having Member with Disability in Household: Identification of households with members with disabilities.  Under 12 Years Member Living in Household: Identification of households with members under 12 years old.  Having Member in Household in Need of Care: Identification of households with members requiring care.  Health Status of the Member of Household: The overall health status of household members.  Having a Smoker Member: Identification of households with members who smoke.  Health Care Utilization by Household Members:  Using Inpatient Services and the Volume of Use by HH Members and Length of Stay: Utilization of inpatient healthcare services, including volume and length of stay.  Using Outpatient Services and the Volume of Use by HH Members: Utilization of outpatient healthcare services, including volume.  Using Dentistry Services by HH Members: Utilization of dental healthcare services.  Using Medicines and Equipment: Utilization of medicines and medical equipment.  Using Diagnostic Services: Utilization of diagnostic services, including pathology, radiology, sonography, radiotherapy, echocardiography, MRI, exercise test, and nuclear medicine.  Using Physiotherapy and Rehabilitation Service: Utilization of physiotherapy and rehabilitation services.  Using Private Services by HH Members: Utilization of private healthcare services.  Health Services Utilization: Overall utilization of various health services.  Utilizing Cancer Treatments: Utilization of healthcare services for cancer treatment.  Utilizing Dialysis Services and the Volume of Use by HH Members: Utilization of dialysis services, including volume.  Utilizing Ambulatory: Utilization of ambulatory healthcare services.  Use of Drug Addiction Cessation Services: Utilization of services for drug addiction cessation.  Health Expenditure Indicators:  Basic Health Insurance Coverage Ratio: The proportion of the population covered by basic health insurance.  Complementary Health Insurance Coverage Status: The status of complementary health insurance coverage.  Inequality in Access (Financial, Geographical, and Cultural) to Healthcare Services and Safe Water: Inequality in access to healthcare services and safe water based on financial, geographical, and cultural factors.  Medical Density: The density of healthcare providers per thousand populations.  Informal Payments or Under-the-Counter Payment: The prevalence of informal payments or under-the-counter payments in healthcare.  Distribution of Income, Education, Skills, Jobs, Opportunities, Physician, Specialized Manpower, Health Expenditures, and Expectations: Distribution of various factors related to income, education, skills, jobs, opportunities, physicians, specialized manpower, health expenditures, and expectations.  Household Health Expenditures: The total health expenditures incurred by households.  Increasing Consumption of Expensive High-Tech Healthcare Services: Trends in the consumption of expensive high-tech healthcare services.  Healthcare Tariff Growth Rate: The growth rate of healthcare tariffs.  Contribution of Healthcare Sector to the Economy: The contribution of the healthcare sector to the overall economy.  Macro-Economic Indicators:  Inflation Rate: The rate of inflation in the economy.  Unemployment Rate: The rate of unemployment in the population.  Economic Growth Rate: The overall economic growth rate.  Employment Status of Household Members: The employment status of household members.  Others:  Percentage of Total Health Expenditure to GDP: The percentage of total health expenditure as a share of the Gross Domestic Product (GDP).  Assumptions and Simplifications:  Standard Definitions: The review assumed the use of standard definitions for variables such as CHE, impoverishment, and healthcare utilization. These definitions were likely consistent with established frameworks in health economics and policy.  Comparability of Data: The review may have assumed that data from different studies were comparable in terms of methodology and measurement, allowing for a meaningful synthesis of results.  Language Inclusion: The review focused on studies published in English and Persian languages, assuming that these languages adequately covered the relevant literature.  Publication Status: Only peer-reviewed journals and publicly accessible grey literature were included, assuming that these sources provide reliable and valid information.  Data Accuracy: The assumption that the data reported in the included studies were accurate and reliable, as the scoping review likely did not involve reanalyzing raw data.  These variables and assumptions collectively provided a comprehensive framework for understanding the complex factors influencing catastrophic healthcare expenditures and impoverishment in Iran. |  |
| Critical appraisal of individual sources of evidence§ | 12 | If done, provide a rationale for conducting a critical appraisal of included sources of evidence; describe the methods used and how this information was used in any data synthesis (if appropriate). | In a scoping review, the emphasis is often on comprehensively identifying and mapping relevant literature rather than critically appraising the quality of individual studies. Critical appraisal is more commonly associated with systematic reviews or meta-analyses where the aim is to synthesize the evidence in a more quantitative and systematic manner. |  |
| Synthesis of results | 13 | Describe the methods of handling and summarizing the data that were charted. | The methods of handling and summarizing the data in the scoping review involve a combination of descriptive statistics and narrative synthesis. Here's a breakdown of the methods:  Data Charting: The information from the included studies was charted using an Excel worksheet and a data extraction form developed by the authors. The charting process involved recording study characteristics and variables relevant to the review question.  Charted Information: The charted information covered various aspects, including study features, key results, and other relevant data points. The data extraction form was developed to capture information such as the source of information, time frame, language, research location, study population, types of interventions, type of studies, and types of articles.  Reviewers and Iterative Process: Two reviewers (M. H, S. G) were involved in the data extraction process, and an iterative approach was taken. The draft table was continuously updated and refined during the conduct of the scoping review. The iterative nature suggests that the process was dynamic, with ongoing adjustments to the data extraction form based on the evolving understanding of the literature.  Synthesis of Results: The analytical framework was used for data synthesis and thematic analysis. The main reviewer (M. H, I. MA, MR. M) extracted and analyzed data from all articles in consultation with other authors. Given the diversity of study designs, descriptive statistics and narrative synthesis were employed.  Descriptive Statistics: Descriptive statistics involve summarizing and presenting the main characteristics of the included studies. These statistics could include averages, percentages, or other quantitative measures that provide an overview of the key findings.  Narrative Synthesis: Narrative synthesis refers to the process of synthesizing information from the included studies in a narrative format. This involves summarizing and interpreting the findings in a qualitative manner, often using text to describe patterns, themes, or relationships between different variables.  Thematic Analysis: Thematic analysis was likely used to identify and explore themes or patterns within the data. This involves categorizing and organizing information into themes to derive meaningful insights from the diverse set of included studies.  Overall, the scoping review employed a combination of quantitative (descriptive statistics) and qualitative (narrative synthesis, thematic analysis) methods to handle and summarize the charted data. The goal was to provide a comprehensive overview of the literature, identify key variables, and present a synthesized narrative of the findings. |  |
| **RESULTS** | | | | |
| Selection of sources of evidence | 14 | Give numbers of sources of evidence screened, assessed for eligibility, and included in the review, with reasons for exclusions at each stage, ideally using a flow diagram. | Identification:  Initial articles identified: 848  Additional articles from Google Scholar: 2  Total articles before removing duplicates: 850  Screening:  After removing duplicates: 769  Excluded based on title and abstract: 500  Reasons for exclusion:  Outcome not related to health equity and financial protection indicators  Lack of information about catastrophic and impoverishing health expenditures and their determinants  Remaining articles after title and abstract screening: 230  Eligibility Assessment:  Fully assessed for eligibility: 149  Excluded after full assessment: 39  Reasons for exclusion not specified  Total articles included in the final review: 110 |  |
| Characteristics of sources of evidence | 15 | For each source of evidence, present characteristics for which data were charted and provide the citations. | Publication Period:  Studies published between 2000 and 2021  Language:  English: 66%  Farsi: 34%  Study Level:  National-level studies: 49%  Provincial-level studies: 51%  Geographical Focus:  28% of provincial-level studies conducted in Tehran  Temporal Distribution:  57% of studies conducted between 2016 and 2021  Study Design:  All studies were observational  Cross-sectional studies: 62%  Data Source:  Primary data: 58%  Secondary data: 42%  Questionnaires Used:  SCI (Statistical Center of Iran) Household Income and Expenditure Survey: 11%  WHO survey: 33%  Self-administered questionnaire: 16%  Sample Size Range:  Ranged from 100 to 1,940,613  Disease Focus:  14 studies focused on diseases  Cancer patients: 5 studies  Dialysis patients: 3 studies  Statistical Models Used:  Logistic random effects regression model: 44% |  |
| Critical appraisal within sources of evidence | 16 | If done, present data on critical appraisal of included sources of evidence (see item 12). | "While critical appraisal of included sources of evidence is a standard component of systematic reviews, we opted not to perform this step in our scoping review. Scoping reviews differ in their primary objectives compared to systematic reviews. The overarching goal of our scoping review was to comprehensively map the existing literature on the variations in catastrophic and impoverishing health expenditures and their determinants in Iran. Scoping reviews are designed to provide an inclusive overview of the available evidence, emphasizing breadth and inclusivity rather than assessing the methodological quality of individual studies. Given the extensive nature of our review and the broad research question, conducting a critical appraisal was considered time-consuming and not aligned with the primary objectives of the scoping review. The emphasis in this review is on identifying the extent, range, and nature of the evidence available, contributing to a comprehensive understanding of the topic." |  |
| Results of individual sources of evidence | 17 | For each included source of evidence, present the relevant data that were charted that relate to the review questions and objectives. | Number of studies assessed: 110  Categories of determinants: Demographic characteristics, Socioeconomic characteristics, Vulnerable persons, Healthcare utilization, Health expenditure indicators, Macroeconomic indicators  Healthcare Needs, Utilization, and Capacity to Pay:  Economic status: Poorest households have the highest CHE incidence (51 studies)  Chronic illness and NCDs: Households with members having chronic illnesses are at higher risk of CHE (19 studies)  Service Utilization: Inpatient and outpatient services contribute to increased CHE (32 studies)  Demographic Factors:  Aging Population: Association between aging population and higher CHE (41 studies)  Household Size: Large households (five or more persons) associated with high CHE (41 studies)  Residency: Residency in rural areas contributes to socioeconomic inequality in CHE (36 studies)  Education and Employment: Education and employment status of the household head impact CHE (20 and 18 studies, respectively)  Health Insurance:  Limited Role of Health Insurance: No significant difference in facing CHE between insured and uninsured individuals  Health Insurance Coverage: Increased from 83% in 2010 to 96% in 2014  Challenges with Health Insurance: Limited benefits packages, co-payment issues in complementary insurance (8 studies)  Comparison with Other Studies:  Consistency with Previous Studies: Findings align with previous studies in Iran and China  International Comparisons: Similar findings in other countries (e.g., Bangladesh, India, Indonesia, Vietnam) regarding factors affecting CHE  Conclusion:  Economic status, chronic illnesses, service utilization, and demographic factors significantly impact CHE in Iran.  Health insurance has a limited role in protecting individuals from CHE, with challenges in benefits coverage and co-payment.  Implications for Scoping Review Objectives:  [Summarize how the presented data contribute to the overall scoping review objectives and the understanding of determinants of CHE in Iran.] |  |
| Synthesis of results | 18 | Summarize and/or present the charting results as they relate to the review questions and objectives. | Incidence and Intensity of Catastrophic Health Expenditure:  National Population Level:  Average Incidence (2000-2020): 3.19%  Range of Incidence: 0.3% to 32.7%  Notable Studies:  Lowest: Hajizadeh et al. in 2003 (0.3%)  Highest: Moradi et al. in 2020 (32.7%)  Primary vs. Secondary Data:  Incidence in Primary Data Studies: 3.83%  Incidence in Secondary Data Studies: 3.37%  Possible Influence of Data Source on Reported Incidence  Provincial Population Level:  Average Incidence: 18.51%  Range of Incidence: 0.4% to 72.7%  Disease-Specific Analysis: Highest CHE observed among households with gastrointestinal cancer patients (72.7%).  Intensity Measures:  Overshoot Intensity: Varied between rural (11.7-19.7%), urban (11.4-20.0%), and all households (0.26-0.65%).  Mean Positive Overshoot (MPO): Ranged from 12.26% to 20.86%, with an average MPO of 12.47%.  Household Impoverishment:  National Level: 3.21% of households impoverished due to health care expenditure.  Provincial Level: 4.78% of households impoverished, ranging from 0.28% to 10.2%.  Financial Protection Indicators:  Fair Financial Contribution Index (FFCI):  Average FFCI: 0.833  Ranged from 0.75 to 0.90  Notable Disparities: Worst FFCI occurred in 2010 (Urban: 0.79, Rural: 0.75), Best in 2007 (Urban: 0.901, Rural: 0.866).  Concentration Index (CI):  Average CI: -0.01  Range: -0.23 to 0.55  Worst CI: Yazdi-Feyzabadi et al. in 2011 (Rural: -0.21, Urban: -0.23)  Kakwani Index:  Average Kakwani Index: -0.149  Worst Kakwani: Rezaei et al. in 2017 (-0.207)  Gini Coefficient:  Average Gini Coefficient: 0.42  Worst GINI: Ghiasvand et al. in 2012 (Rural: 0.52, Urban: 0.52)  Determinants of Catastrophic Health Expenditure:  Socioeconomic Characteristics:  Economic status, household settlement (urban/rural), health insurance status, wealth index, homeownership, and insurance scheme affiliation identified as key determinants.  Demographic Characteristics:  Household size, gender of the household head, education level of the household head, employment status of the household head, age, and marital status of the household head considered as significant factors.  Vulnerable Persons in Household:  Aging population, presence of elderly members, households with members under 5 years, chronic illness, disability, and need for care are strongly associated with CHE.  Healthcare Utilization:  Inpatient and outpatient services, dental care, medicines and equipment, diagnostic services, physiotherapy, rehabilitation, and utilization of cancer treatments and dialysis services contribute to CHE.  Health Expenditure Indicators:  Limited basic health insurance coverage, challenges with complementary health insurance, financial burden, geographical inaccessibility, and cultural barriers contribute to CHE.  Macroeconomic Indicators:  OOP health expenditures positively correlated with macroeconomic profiles such as inflation rate, GDP per capita, budget deficit, illiteracy rate, and more. |  |
| **DISCUSSION** | | | | |
| Summary of evidence | 19 | Summarize the main results (including an overview of concepts, themes, and types of evidence available), link to the review questions and objectives, and consider the relevance to key groups. | This scoping review provides a comprehensive overview of the incidence, intensity, and determinants of catastrophic health expenditure (CHE) in Iran, as well as the associated financial protection indicators. The study spans the period from 2000 to 2020 and examines national and provincial levels, considering various thresholds and methodologies used across 112 studies.  Incidence of Catastrophic Health Expenditure (CHE):  At the national level, the average incidence of CHE was estimated to be 3.19%.  Different thresholds were used, with 40% of nonfood expenditure being the most commonly employed.  The lowest reported CHE percentage was 0.3%, while the highest was 32.7%, indicating considerable variability.  Incidence at the provincial level was higher, with an average of 18.51%.  Disease-specific analysis revealed a 35% incidence of CHE, with variations ranging from 3.37% to 72.7%.  CHE intensity, measured by overshoot and mean positive overshoot (MPO), averaged 10.1% and 12.47%, respectively, at the national level.  Impoverishment due to Health Expenditure:  About 3.21% of households at the national level fell below the poverty line due to healthcare expenses.  The impoverishing health expenditure varied across rural and urban areas, ranging from 0.9% to 11.5%.  Financial Protection Indicators:  Fair Financial Contribution Index (FFCI) averaged 0.833, indicating less-than-optimal fairness in health financing.  Concentration Index (CI) averaged -0.01, suggesting that OOP payments were more concentrated in poor households.  Kakwani index averaged -0.149, indicating regressive financing in healthcare.  Gini coefficient averaged 0.42, signaling inequality in health resources distribution.  Trends and Policy Impact:  Despite the Health Transformation Plan in 2014, which aimed to reduce out-of-pocket (OOP) payments, CHE levels increased to 3.7% in 2020.  Studies using WHO questionnaires reported higher CHE levels compared to those using the Household Income and Expenditure Survey (HIES).  Determinants of Catastrophic Health Expenditure:  Six categories of drivers were identified: demographic characteristics, socioeconomic characteristics, vulnerable persons, healthcare utilization, health expenditure indicators, and macroeconomic indicators.  Economic status, healthcare needs, service utilization, large household size, rural residency, and low education level were identified as major drivers.  Health insurance, while improving in coverage, showed limited effectiveness in protecting against CHE, with issues such as low coverage and high co-payments in complementary insurance.  Relevance to Key Groups:  The study's findings are relevant to policymakers, researchers, and public health practitioners involved in assessing and improving financial protection in the Iranian healthcare system.  The identification of specific determinants and the impact of policy interventions provides valuable insights for addressing and reducing CHE.  The study highlights the ongoing challenges in achieving universal health coverage and the need for targeted strategies to protect vulnerable populations.  Link to Review Questions and Objectives:  The study directly addresses the objectives of understanding the trends, variations, and determinants of CHE in Iran, providing a comprehensive synthesis of evidence over the specified period.  It assesses the impact of policy measures, such as the Health Transformation Plan, on CHE incidence and explores the implications for financial protection indicators.  In summary, this scoping review contributes a detailed and nuanced understanding of the landscape of catastrophic health expenditure in Iran, shedding light on the multifaceted factors influencing financial protection and highlighting areas for targeted interventions and further research. |  |
| Limitations | 20 | Discuss the limitations of the scoping review process. | The scoping review process, while valuable in providing a broad overview of the landscape of catastrophic health expenditure (CHE) in Iran, is not without its limitations. Here, we discuss some of the key limitations of the scoping review:  Heterogeneity in Study Designs and Data Sources:  The inclusion of studies with different designs (national household surveys, provincial household surveys, targeted population surveys) introduces variability in methodologies, sample sizes, and data collection instruments. This heterogeneity can affect the comparability and generalizability of findings.  Variation in Thresholds for Defining CHE:  The application of different thresholds to define catastrophic health expenditure in the included studies contributes to the variability in reported incidence rates. The choice of threshold, such as 40% of nonfood expenditure, influences the proportion of households classified as experiencing CHE, making cross-study comparisons challenging.  Diverse Measures and Outcomes:  The scoping review captures a wide range of outcomes, including incidence, intensity, and impoverishment due to health expenditure. While this breadth is informative, it also means that the studies may not be directly comparable due to variations in outcome measures and definitions.  Temporal and Geographic Variability:  The scope of studies spans a considerable time period (2000 to 2020), during which health policies and economic conditions may have changed. Temporal variations could impact the relevance of findings to the current context. Additionally, regional differences might exist, given the diversity in provinces.  Imbalance in Focus on CHE vs. Impoverishment:  The primary focus of the studies on catastrophic health expenditure rather than impoverishment introduces a potential limitation. While some studies indirectly address impoverishment, the lack of a specific focus on this aspect may limit the depth of understanding of the economic impact of health expenses.  Potential Publication Bias:  The review acknowledges the possibility of missing studies, particularly those classified as 'grey literature.' This may introduce a publication bias, as studies with non-significant findings or those outside conventional publication channels may not be adequately represented.  Lack of Uniformity in Poverty Line Definitions:  The use of different poverty lines (subsistence, national, international) to measure impoverishment adds another layer of complexity. Variations in poverty line definitions may impact the comparability of findings and limit the precision of estimates.  Incomplete Assessment of Grey Literature:  The review notes that some studies classified as 'grey literature' might be missing. The exclusion of relevant non-peer-reviewed sources could lead to an incomplete representation of available evidence.  Absence of a Meta-analysis:  The scoping review does not include a meta-analysis, which could have provided a quantitative synthesis of results. The absence of a meta-analysis limits the ability to quantitatively summarize the overall impact and magnitude of catastrophic health expenditure in Iran.  Interpretation and Policy Implications:  While the study provides evidence for discussions on policy and health financing reform, the interpretation and specific policy implications might be limited by the heterogeneity of the included studies.  Despite these limitations, the scoping review serves as a valuable resource for understanding the current state of catastrophic health expenditure in Iran and identifying areas for further research and policy development. It provides a foundation for future systematic reviews or meta-analyses to delve deeper into specific aspects of the topic. |  |
| Conclusions | 21 | Provide a general interpretation of the results with respect to the review questions and objectives, as well as potential implications and/or next steps. | The results of the scoping review on catastrophic health expenditure (CHE) in Iran have significant implications for health policy, financial protection, and the pursuit of Universal Health Coverage (UHC). Here is a general interpretation of the results and their implications:  Ineffectiveness of Basic Health Insurance:  The study highlights a critical finding that basic health insurance in Iran is not effectively reducing catastrophic health expenditures. This underscores the need for a more comprehensive and integrated approach to health financing to ensure that people not only have access to services but are also protected from financial hardships associated with healthcare costs.  Call for Integrated Reform Strategy:  The study advocates for a more integrated reform strategy that enhances the breadth, depth, and height of insurance coverage. This suggests that reforms should not only focus on expanding coverage but also on improving the comprehensiveness of benefits packages and minimizing out-of-pocket spending.  Long-Term Integration of Insurance Schemes:  The call for the integration and harmonization of various insurance schemes in the long run emphasizes the importance of a unified and coordinated approach to health financing. Policymakers are urged to consider the design of benefits packages and the level of cost-sharing, with particular attention to protecting vulnerable populations.  Flexible Short-Term Responses:  Acknowledging the need for short-term responses based on the social and political context of the country suggests a pragmatic approach to health financing reforms. Flexibility is essential in addressing immediate challenges while working towards the long-term goal of achieving financial protection and reducing catastrophic health expenditures.  Importance of Monitoring and Evaluation:  The study underscores the importance of systematic monitoring of catastrophic health expenditures. Continuous monitoring will provide valuable data for evidence-based policymaking, allowing for adjustments and refinements in health financing policies over time.  Government Contribution and Tax Funding:  The recommendation for the government of Iran to consider increasing its contribution to the health sector through tax funding is significant. Reducing dependency on out-of-pocket payments requires sustainable and sufficient funding from public sources to ensure equitable access to healthcare services.  Increased Interest and Need for Longitudinal Studies:  The review acknowledges the growing interest in studies assessing CHE in Iran over time. However, it highlights the predominance of cross-sectional studies and calls for more longitudinal research. Longitudinal studies would provide a deeper understanding of trends, enabling more robust analyses of the factors influencing catastrophic health expenditures and impoverishment.  In summary, the study emphasizes the urgency of addressing the financial vulnerabilities associated with healthcare costs in Iran. Moving forward, policymakers are encouraged to consider a holistic reform strategy, combining short-term measures with long-term integration of insurance schemes and sustained government commitment to health funding. Additionally, the need for ongoing research, particularly longitudinal studies, is crucial for continuously monitoring and adapting health financing policies to meet the evolving needs of the population. |  |
| **FUNDING** | | | | |
| Funding | 22 | Describe sources of funding for the included sources of evidence, as well as sources of funding for the scoping review. Describe the role of the funders of the scoping review. | The scoping review conducted does not have any external funding from specific funders. The review was undertaken as part of the authors' academic without financial support from external organizations or grants. |  |

### **Supplementary 6. Interview Guide**

Main Questions

Name and Last Name

Educational Level

Current Job Title and Position

Work Experience in Health Policy and Healthcare Cost Containment

Work Experience in Futures Studies and Future Outlook

Identifying Challenges in Healthcare Cost Containment and Health Poverty:

How do you currently evaluate the state of the healthcare system in terms of cost containment and health poverty?

In your opinion, what are the most significant weaknesses, strengths, and challenges faced by households in dealing with healthcare cost containment and health poverty in the country?

Trend Analysis

What social issues and trends will be influential in the future of healthcare cost containment and health poverty?

What economic issues and trends will be influential in the future of healthcare cost containment and health poverty?

What technological issues and trends will be influential in the future of healthcare cost containment and health poverty?

What political issues and trends will be influential in the future of healthcare cost containment and health poverty?

In response to the above trends, what actions should be prioritized?

What do you consider the priorities and key drivers for the future of healthcare cost containment and health poverty?

Mind Models

In your opinion, what model should be adopted in the future for healthcare cost containment and health poverty in Iran?

What is the ideal future for healthcare cost containment and health poverty?

What actions should be taken now to achieve this ideal future?

What are the highest priority actions?

### **Supplementary 7. Hypothetical sample of cross-impact matrix**

|  | V1 | V2 | V3 | V4 | V5 |
| --- | --- | --- | --- | --- | --- |
| V1 | 0 | 1 | 0 | 1 | 3 |
| V2 | 3 | 0 | 1 | 1 | 1 |
| V3 | 0 | 0 | 0 | 0 | 2 |
| V4 | 0 | 0 | 2 | 0 | 0 |
| V5 | 3 | 0 | 2 | 0 | 0 |

### **Supplementary 8. Summary Characteristics of Included Studies**

| **The data extraction incidence and intensity of CHE, % (National population level)** | | | | | | | | | |
| --- | --- | --- | --- | --- | --- | --- | --- | --- | --- |
| **No.** | **First author (year)** | **Objective(s)** | **Study design** | **methodology** | **Has Logit Mode** | **Data collection method** | **Publication Type/ language** | **Years of data collection** | **Study population** |
| 1 | Razavi, S et al. 2005 ([23](#_ENREF_23)) | Measuring equity in household’s health care payments according to fairness in financial contribution (FFC) 1995 to 2002. | cross-sectional study | WHO method | NO | SCI questionnaire | Journal Article- Persian | 1995 to 2002 | Iranian households |
| 2 | Hanjani, HM et al. 2006 ([24](#_ENREF_24)) | A performance assessment of health system based on its financial function. | cross-sectional study | WHO method | Yes | Secondary data | Journal Article- Persian | 2002 | Iranian households |
| Determinants of exposure to CHE: insurance status(-), urbanization(-), The age of the head of the household (over 65+)(+), Household settlement (rural+), household head literacy level (+), household head employment(-), household size (less than 5 member)(-), household head gender(men)(+), Marital status of the head of the household(-) | | | | | | | | | |
| 3 | Mehrara,Mohsen et al. 2010 ([25](#_ENREF_25)) | Investigated the extent of catastrophic health expenditure as a first step to developing appropriate policy responses. | descriptive-analytic cross-sectional study | WHO method | Yes | Secondary data | Journal Article- Persian | 2003-2007 | Iranian households |
| Determinants of exposure to CHE: rural families (+), Per capita household housing infrastructure(-), the families with children below 12 years and old above 60 year(+), the families who have no insurance(+), employment of the head of the household(-), Number of working members in the household(-), marital status(-), wealth index or the quantile in which the household is located(-), equivalent household(-). | | | | | | | | | |
| 4 | Masaeli, Arashk et al. 2011 ([26](#_ENREF_26)) | Determine the extent of high health costs, and catastrophic and impoverishment expenditures. | Analytical research | WHO method | No | WHO questionnaire | Journal Article- Persian | 2011 | Iranian households |
| Determinants of exposure to CHE: the economic status(-), households with a chronic patient(+). | | | | | | | | | |
| 5 | Razavi, Seyed Moaven et al. 2011 ([27](#_ENREF_27)) | Employed multivariate analysis regressions (Probit), with the catastrophic payment event as a binary dependent variable, based on data from HIES for 1995 (pre-SAP) and 2002 (post-SAP). | cross-sectional study | WHO method Probit models | Yes | WHO questionnaire | Original Article/ English | 1995 vs 2002 | Iranian households |
| 6 | Hajizadeh, M et al. 2011 ([28](#_ENREF_28)) | Provide a greater understanding about the inequality and determinants of the OOPE and the CHE for hospital services in Iran using a nationwide survey data | Cross-sectional study | WHO method Concentration index Heckman selection model | No | Secondary data | Original Article/ English | 2003 | Inpatient services in Iran |
| Determinants of exposure to CHE: length of stay(+), admission to a hospital owned by private sector(+) or Ministry of Health and Medical Education(-), lower household wealth index(+), and living in remote areas(+), being self-employed(-), male ratio of household(-) , education level(-), household size(+), health insurance coverage(-) | | | | | | | | | |
| 7 | Nekoei Moghadam, M et al. 2012 ([29](#_ENREF_29)) | Measure percentage of Iranian households exposed to CHE and to explore its determinants. | Cross-sectional basis descriptive-analytica | WHO method | Yes | Secondary data | Original Article/ English | 2008 | Iranian households |
| Determinants of exposure to CHE: Utilizing ambulatory, hospital, and drug addiction cessation services as well as consuming pharmaceuticals(+), health insurance coverage(-), household size(+), and economic status(-), use of outpatient service(+). | | | | | | | | | |
| 8 | Soofi, M et al. 2013 ([30](#_ENREF_30)) | Measure Iranian households' exposure to CHE and surveying the factors affecting this expenditure. | cross-sectional study | WHO method | Yes | Secondary data | Journal Article- Persian | 2001 | Iranian households |
| Determinants of exposure to CHE: having a family member suffering from a chronic disease(+), member in need of care(+), family`s financial condition(-), and living in rural areas(+), health insurance(-), use of outpatient service(+). | | | | | | | | | |
| 9 | Raghfar, H et al. 2013 ([31](#_ENREF_31)) | Assess the fair financial contribution index and influencing factors in the rate of households in nine regions in Iran. | Longitudinal study | WHO method | NO | Secondary data | Journal Article- Persian | 1984-2010 | Iranian households |
| Determinants of exposure to CHE: Age and number of household members(+), inpatient services(+), dental services(+). | | | | | | | | | |
| 10 | Abolhallaje, M. et al. 2013 ([32](#_ENREF_32)) | Identify measures of fair financing of health services and determinants of fair financing contribution, regarding the required share of households that prevents their catastrophic payments. | cross-sectional analytical study | Statistical analysis | NO | Secondary data | Original Article/ English | 2002-2005-2008 | Iranian households. |
| Determinants of exposure to CHE: quality/social determinants: employment situation of the head of family(-), no(-)/low(-)/high(+) education of the head, sex of the head(male+), age of the head(+), number of the members of family(+), number of the members over 60(+), number of kids below 12(+), number of the employed persons in family(-), having health insurance(-), large housing(-)/ socio-economic indicators (in the earlier studies): out-of-pocket share in total expenditure(+), horizontal & vertical inequality indicators(+), health financing distribution indicators of FFCI(+)/ measurement indicators: out-of-pocket changing rules and indicators(+), households’ willingness to pay for health services(+), sources of growth in OOP and prepayment funds(+), contingent valuing of health insurance premium(+), differences in health payments among different deciles in urban and rural areas(+), links between health & other essential payments among different deciles in urban and rural areas(+), number of the uninsured in the informal sector(+), needs for special programs in government(+) budget to support the uninsured(+), needs for health insurance rules of managed care(+), needs for health insurance contracts with private providers(+), households’ socio-economic status(+), equality/inequality conditions of the distribution of risk of financing(+), and economic aspects of health expenditure distribution(-), high inflation rates in the health sector and in the average for total consumption expenditure (+), growing the numbers of physicians and other educated health workers (+), increasing the number of insured people from less than 20 million urban residents to more than 80 % of the total population (+), most of the payment by the public in OOP sources goes to services from the private sector and for under-the-counter payment for services covered by the insurance organizations (+), unsatisfaction by the public sector services or the health insurance support (+), lack of preventing the private medical persons to work out of the regulated tariff rules or to ignore the insurance organization rules easily (+), inefficient social health insurance mechanism to reduce the direct payments from households (+), lack of well-organized services by the public sector hospitals and clinics (+),Baumol variable. | | | | | | | | | |
| 11 | Samadi, A et al. 2013 ([33](#_ENREF_33)) | Surveyed the determinants of health expenditures in Economic Cooperation Organization (ECO) countries. | descriptive-analytic cross-sectional study | Panel data econometrics methods | NO | Secondary data | Original Article/ English | 1995-2009 | ECO countries include Iranian households |
| Determinants of exposure to CHE: Health expenditures per capita and GDP per capita(-), the proportion of population below 15 and above 65 years old(+), number of physicians(-), and urbanization(-). | | | | | | | | | |
| 12 | Zare, Hossein et al. 2014 ([34](#_ENREF_34)) | An inequalities assessment of health care expenditures in Iran. | A longitudinal study | WHO method | NO | SCI questionnaire | Journal Article- Persian | 1984–2010 | Iranian households |
| 13 | Mohammadzadeh Y et al. 2014 ([35](#_ENREF_35)) | Evaluate the impact of household socio-economic status on the probability of facing with impoverishing health expenditure | Retrospective descriptive study | Logit Technique | Yes | Secondary data | Journal Article- Persian | 2007 to 2011 | Iranian households |
| Determinants of exposure to CHE: the employment of household head(-), homeownership(-), the most educated people in the family(-), more per capita area of residence(-), family being in high income deciles(-), insurance coverage and increases with a growth in household size(-), living in more developed provinces(-). | | | | | | | | | |
| 14 | Ahmadi, AM et al. 2014 ([36](#_ENREF_36)) | Assessing the factors affecting in household OOP payments in health system of Iran and using a two part model for assessing these factors | descriptive-analytic cross-sectional study | Multi-stage method, Regression model | Yes | Secondary data | Journal Article- Persian | 2005-2010 | Iranian households. |
| Determinants of exposure to CHE: the economic status(+), the elderly(+), household dimension(+), urbanization and not having insurance coverage(+) | | | | | | | | | |
| 15 | Fazaeli, Ali Akbar et al. 2015 ([37](#_ENREF_37)) | Determination of main factors on catastrophic health expenditures in Iranian households. | descriptive-analytic cross-sectional study | WHO method | Yes | Secondary data | Original Article/ English | 2010 | Iranian households |
| Determinants of exposure to CHE: rural households(+), the number of individuals older than 65 years in each household(+), the number of individuals younger than 5 years(+), illiterate or low literacy householder(+), employed householder status(-), the number of employed persons in household(-), insured household status(-), Gender of the head of the household(female+), presents equivalent household size(–), household expenditures increases nonlinearly(+), increase of the number of household member(+), Marital status of the head of the household(-), Number of household expenditure deciles(+). | | | | | | | | | |
| 16 | Ghiasvand, H et al. 2015 ([38](#_ENREF_38)) | Investigate 3 objectives: First, the mean of OOP among Iranian households for health services; second, the headcount and overshoot measures of CHE; and finally the level of inequality in its distribution. | Descriptive-analytical | WHO method | NO | Secondary data | Original Article/ English | 2013 to 2014 | Iranian households |
| 17 | Fazaeli, Amir Abbas et al. 2015 ([39](#_ENREF_39)) | Present a trend analysis for the indicators related to fairness in healthcare’s financial burden in rural and urban population of Iran. | A time trend study | WHO method | NO | Secondary data | Original Article/ English | 2003 to 2010 | Iranian households |
| 18 | Masaeli,Arashk et al. 2015 ([40](#_ENREF_40)) | Determine the extent of high health costs, and catastrophic and impoverishment expenditures for informed policy making. | descriptive-analytical study | WHO method | NO | Secondary data | Journal Article- Persian | 2011 | Iranian households |
| Determinants of exposure to CHE: the economic status(-), households with a chronic patient(+). | | | | | | | | | |
| 19 | Yousefi, Mehdi et al. 2015 ([41](#_ENREF_41)) | Determine and present some indices of household financial contribution in health system in Iran. | descriptive-analytic cross-sectional study | WHO method | NO | Secondary data | Journal Article- Persian | 2011 | Iranians households |
| 20 | Rezaei, Satar et al. 2015 ([42](#_ENREF_42)) | Determine the impact of some of the key explanatory variables on household healthcare expenditures across the provinces of Iran. | cross-sectional study | Panel data econometric model, F-Limer and Hausman, | NO | Secondary data | Original Article/ English | 2006 to 2013 | Iranian household |
| Determinants of exposure to CHE: household healthcare expenditures per capita(+), number of physicians per 10,000 population(+), the degree of urbanization(+), the proportion of the population that was 65 or older(+), household income per capita(-), and literacy rate(+) | | | | | | | | | |
| 21 | Ghiasvand, H. et al. 2015 ([43](#_ENREF_43)) | Investigated the Iranian rural and urban households’ inequality in payments on food and OOP health expenditures from 1998 to 2012. | A cross-sectional time trend study | Gini Coefficients, Concentration and Kakwani indices | NO | Secondary data | Original Article/ English | 1998 to 2012. | Iranian rural and urban |
| Determinants of exposure to CHE: gender of the household’s head, health status of the member of household, the size of household, residency in Tehran city, number of previous hospitalization, having a house, the level of income and finally complementary health insurance coverage. | | | | | | | | | |
| 22 | Fazaeli, A. A et al. 2016 ([44](#_ENREF_44)) | Illustrating the consequences of Iranian household to health system financial contribution in terms of burden and incomes approaches. | cross-sectional study | WHO and World Bank | NO | SCI questionnaire | Original Article/ English | 2012 | Iranian households |
| Determinants of exposure to CHE: insurance status(-), urbanization(+). | | | | | | | | | |
| 23 | Amin, E. et al. 2016 ([45](#_ENREF_45)) | Explore the impact of independent variables that had a direct relationship with household economic status (household total expenditure and insurance expenditure) and household access to health services (distribution of physicians over the household) in an urban and rural area. | Retrospective longitudinal study | OLS Regression technique Panel dataset | Yes | Secondary data | Journal Article- Persian | 1981 to 2011 | Iranian households of 24 section |
| Determinants of exposure to CHE: household economic status (household total expenditure (+) and insurance expenditure (+)), household access to health service (distribution of physician over the household (+)) in urban and rural area, demographic characteristics (proportion of elderly over the household(+)), technological improvement(-), live in urban areas(+), Failure in the rules of economic evaluation(+), Inefficiency of the insurance system(+), Weakness in service delivery and surveillance system(+) | | | | | | | | | |
| 24 | mohammad alizadeh et al. 2016 ([46](#_ENREF_46)) | Identify the robust determinants of health sector costs in Iran under the uncertainty of the model. | Retrospective descriptive study | Bayesian Averaging of Classical Estimates (BACE) | NO | Secondary data | Journal Article- Persian | during 1979-2013 | Iranian households |
| Determinants of exposure to CHE: Per capita income(-), urbanization rate(+), per capita public health costs(-), dependency ratio(+), physician per capita(+), and unemployment rate(+). | | | | | | | | | |
| 25 | Rezaei, Satar et al. 2016 ([47](#_ENREF_47)) | Examine the determinants of healthcare spending in Iran over the periods of 1978-2011. | Retrospective descriptive study | A time series analysis, Autoregressive distributed lag approach Error correction method | NO | Secondary data | Original Article/ English | 1978 to 2011 | Iranian household |
| Determinants of exposure to CHE: GDP per capita(+), illiteracy rate(+), degree of urbanization(+), population aging(+), total number of physician per 10,000 populations(-), literacy(+), advancement of new technology(+), the costs of dying and time-to-death(+), | | | | | | | | | |
| 26 | Rad, E. H et al. 2016 ([48](#_ENREF_48)) | Assess the taxation system and health insurance contribution of Iranians. | descriptive- analytical study | Data survey, Kakwani index, A regression model | NO | Secondary data | Original Article/ English | 2012 | Iranian household |
| Determinants of exposure to CHE: persons older than 65 years old(+), urbanization(+), income status(-), employing status(-), literacy(+). | | | | | | | | | |
| 27 | Vahid Yazdi-Feyzabadi et al. 2017 ([49](#_ENREF_49)) | Measure the percentage of households impoverished due to OOP payments in Iran provinces | Retrospective descriptive study | Mann-Whitney U test and descriptive statistics | NO | Secondary data | Journal Article- Persian | 2008-2014 | Iranian households |
| Determinants of exposure to CHE: Burden of chronic diseases(+), Lack of adequate health insurance at the time of illness in terms of the method of payment to the provider(+), the amount of coverage of treatment costs (+), the type of service package committed and the population covered (+), Existence of elderly people in the family (+), Living in the rural(+), Per capita household expenditure(+), disability (+), 7.Inadequate geographical distribution of specialized manpower. | | | | | | | | | |
| 28 | Yazdi Feyzabadi, V, 2017 ([50](#_ENREF_50)) | Measure the incidence and intensity of CHE in Iranian provinces 2008-2014. | Retrospective study | Data surveys, Descriptive statistics , Mann-Whitney U test, and index of disparity (ID) | NO | Secondary data | Journal Article- Persian | 2008-2014. | Iranian provinces |
| Determinants of exposure to CHE: Socioeconomic status(+),inequality in access to health services(+), incidence of chronic and incurable diseases and disabilities(+),Scheme on health service utilization (+),Unemployment(+), Per capita income and its distribution(+), Inflation rate(+). | | | | | | | | | |
| 29 | Nouraei Motlagh, S et al. 2017 ([51](#_ENREF_51)) | Investigate affecting factors on probability of households facing to CHE, estimate FFCI and Gini indices in deprived states of Iran. | descriptive analytical study | Bayesian econometrics model | Yes | Secondary data | Journal Article- Persian | 2012 | Iranian households |
| Determinants of exposure to CHE: dentistry and hospital services(+), Increased number of family members with >65 year old(+), low literacy and unemployed parent(+), female guardian and without insurance coverage(+), expenditure deciles(+), inpatient service(+), equivalent households size(+), gender of the head of household (female)(+), living in the urban(+). | | | | | | | | | |
| 30 | Homaie Rad, Enayatollah et al. 2017 ([52](#_ENREF_52)) | Test the hypothesis “CHE increase the probability of retirees to go back to work.” | cross-sectional study | Xu method | Yes | Secondary data | Original Article/ English | 2012 | Iranian households who  have been receiving retirement pension |
| Determinants of exposure to CHE: Chronic diseases like cancer(+), renal diseases(+), and cardiovascular diseases(+), increase in household size(+), households headed by male retirees(+). | | | | | | | | | |
| 31 | Ali Mohammad Ahmadi et al. 2017 ([53](#_ENREF_53)) | Analyze the impact of family’s socio-economic status, and government health policies on different levels of health expenditures of households in Iran. | descriptive-analytic cross-sectional study | Probit, model, Data analysis | NO | SCI questionnaire | Journal Article/ Persian | 2014 | Iranian households |
| Determinants of exposure to CHE: Householder education level(-), Age(+), Gender (male householders(+), Per capita income(+), Size of household(+), health insurance coverage(-),households with rural insurance(-), social security insurance(-), complementary insurance(-) and medical treatment insurance(-),households with better socioeconomic situation (-). | | | | | | | | | |
| 32 | Fazaeli, Ali Akbar et al. 2017 ([54](#_ENREF_54)) | Investigated the financial participation of Iranian urban households in the health sector before and after the development plan. | Cross-sectional retrospective study before and after analysis | Data survey, Descriptive statistics, Data analyzes |  | Secondary data | Journal Article- Persian | 2004-2016 | Iranian households in urban |
| Determinants of exposure to CHE: inflation rate(+), health transformation plan in 2014(-). | | | | | | | | | |
| 33 | Vahid Yazdi-Feyzabadi et al. 2018 ([55](#_ENREF_55)) | Estimate the prevalence and intensity of CHE and investigate main factors that influence the probability of CHE in Iran. | Retrospective descriptive study | WHO method | Yes | SCI questionnaire | Original Article/ English | 2008 to 2015 | Iranian households |
| Determinants of exposure to CHE: Expenditure quintiles (+), Household settlement(rural+), Household head gender(female+), Hospitalized person in household(+), Household using outpatient care(+), + 60 member living in Household(+), higher income(-), receiving inpatient services(+),Interestingly, limited geographical and cultural accessibility (-). | | | | | | | | | |
| 34 | Ghiasvand, H et al. 2018 ([56](#_ENREF_56)) | Present a clear picture of the financial protection situation in Iran from 2003-2014. | cross-sectional study | WHO method | Yes | Secondary data | Original Article/ English | 2003-2014 | Iranian households |
| Determinants of exposure to CHE: Living in rural regions(+), having literate heads(+), owning a house,(-) living in a rental house(+), and placing in higher total expenditures quartiles(+). | | | | | | | | | |
| 35 | Moradi, T et al. 2018 ([57](#_ENREF_57)) | Decompose inequality in financial protection of Iranian households after the implementation of the Health Transformation Plan. | cross-sectional study | WHO method | Yes | Secondary data | Original Article/ English | 2015 to 2016 | Iranian households |
| Determinants of exposure to CHE: Economic status(-), Education level of household head(-), access to healthcare services(+), household size(+), age of household head(+), the gender of household head(female+), outpatient services such as diagnostic services(+), dentistry(+), rehabilitation(+), outpatient consultations(+), medicines and equipment (+). | | | | | | | | | |
| 36 | Behzadifar, M. et al. 2018 ([58](#_ENREF_58)) | Evaluate the temporal pattern of OOP expenditures related to Iranian healthcare services during 1995–2014. | Longitudinal study | Data collection, Trend analysis by an exhaustive and comprehensive review. | NO | Secondary data | Original Article/ English | 1995–2014 | Iranian households |
| Determinants of exposure to CHE: patient gender (males+), diagnostic services(+) | | | | | | | | | |
| 37 | Assari Arani, A et al. 2018 ([59](#_ENREF_59)) | Evaluated the effects of the plan on health equity indices. | cross-sectional study | WHO method | NO | Self-administered questionnaire. | Original Article/ English | Dec 2015 to mid-Mar 2017. | Iranian households |
| 38 | Fazaeli, A. A. et al.2018 ([60](#_ENREF_60)) | Calculated financial contribution of people in Iran health system in 2015. | descriptive analytical study | Statistical analysis | NO | Secondary data | Journal Article- Persian | 2015 | Iranian households |
| Determinants of exposure to CHE: using dental care(+), using medical services or diagnosis(+) | | | | | | | | | |
| 39 | Yazdi-Feyzabadi Vahid et al. 2019 ([61](#_ENREF_61)) | Investigate the occurrence, intensity and inequality in distribution of CHE in the years before and after HTP. | cross-sectional survey before and after analysis | WHO method World Bank method | No | Secondary data | Original Article/ English | 2011 to 2017 (before and after HTP) | Iranian households |
| 40 | Rezaei Satar  et al. 2019 ([62](#_ENREF_62)) | Quantify socioeconomic inequality in facing CHE and to identify the main factors contributing to socioeconomic inequality in CHE in Iran. | Retrospective descriptive study | WHO method | Yes | SCI questionnaire | Original Article/ English | 2017 | Iranian households |
| Determinants of exposure to CHE: Household size(-); age(+), sex(female+), and educational status of the head of household(-); having a senior member (over 65 years) or a child member (5 years or younger) of the household(+); residential place of the household (rural+ vs. urban-); province(Tehran+); health insurance coverage(-); use of inpatient care(+), dental care(+), and outpatient care(+); and the wealth index of the household(-). | | | | | | | | | |
| 41 | Ahmadnezhad, E et al. 2019 ([63](#_ENREF_63)) | Investigate the impact of the HTP on the level and pattern of OOP payments for health care. | descriptive-analytic cross-sectional study | WHO method | NO | Secondary data | Original Article/ English | 2013 and 2016 | Iranian households |
| Determinants of exposure to CHE: medicines (+) | | | | | | | | | |
| 42 | Kheibari, M. J et al. 2019 ([64](#_ENREF_64)) | Assessed the reform by changes in variables representing distribution of health payments and CHE. | descriptive-analytic cross-sectional study | WHO method | NO | SCI questionnaire | Original Article/ English | 2010 to 2016 | Iranian households |
| 43 | Amiresmaili, Mohammadreza et al. 2019 ([65](#_ENREF_65)) | Calculated the population at risk of facing catastrophic expenditure due to purchasing three selected medicines (metformin, atorvastatin and amoxicillin) in Iran. | cross-sectional study | WHO method | NO | Secondary data | Original Article/ English | 2013 | Iranian households |
| 44 | Masoudi Asl, Iravan et al. 2019 ([66](#_ENREF_66)) | Investigating the health costs trend in Iran and the policies adopted to manage them better. | descriptive-analytical study | General review, Semi-structured interviews, Data analysis, Thematic framework | NO | Secondary data | Journal Article- Persian | 2002-2013 | Iranian households |
| Determinants of exposure to CHE: NCDs disease(+), chronic disease(+), private outpatient health services(+), Tariff increase(+), Lack of financial protection(+), Low density of health care providers in disadvantaged areas(+),Expand public sector services to disadvantaged areas(-), Adoption of public insurance law(-), Free insurance for rural(-), Multiplication of basic insurance funds(+), reduction of accumulation of insurance resources(+), a high number of people without health insurance(+), Currency price unification policy(+), Rising exchange rates and high inflation(+), The financial burden of targeted subsidy plan(+), Migration from rural to urban areas and increasing marginalization and increasing the number of uninsured due to these migrations(+), Increasing the level of public awareness and creating consumers induced demand(+), Change of consumption towards branded drugs(+), supplier induced demand(+) and performing unnecessary diagnostic and therapeutic interventions(+), Lifestyle changes and adopting a lifestyle pattern associated with high-risk health behaviors(+). | | | | | | | | | |
| 45 | Shabani, Hamed et al. 2019 ([67](#_ENREF_67)) | Investigate the determinants of health expenditures in Iran and the other member countries which are the members of Perspective Documents of 1404 (Hijri) of Iran. | descriptive-analytic study | Panel data from World Bank. | NO | Secondary data | Journal Article- Persian | 1995 to 2014 | Iranian households |
| Determinants of exposure to CHE: GDP per capita(+), urbanization rate(+), the percentage of the population older than 65 years and above(+), NCDs and chronic disease(+). | | | | | | | | | |
| 46 | Pakdaman, Mohsen et al. 2019 ([68](#_ENREF_68)) | Determine the effect of macroeconomic indicators on health expenditure. | descriptive analytical study | Time series models in econometrics, Vector Auto Regression, Granger causality technique. | NO | Secondary data | Original Article/ English | 1995–2014 | Iranian households |
| Determinants of exposure to CHE: gross domestic production (GDP)(+), gross national production(+), national income(+), and national consumption countries income(+), Unemployment(+), liquidity rate(+), inflation rate(+), budget deficit(+), population rate(+). | | | | | | | | | |
| 47 | Rezaei, Satar et al. 2020 ([69](#_ENREF_69)) | Measure equity in OOP payments for healthcare and the incidence of CHE among Iranian households over time. | Retrospective descriptive study (measurement) | WHO method Trend series regression. | NO | Self-administered questionnaire. | Original Article/ English | 1991 to 2017 | Iranian households |
| 48 | Abdi,ZH et al. 2020 ([70](#_ENREF_70)) | Undertake a descriptive analysis of changes in health spending associated with implementation of the latest health sector reform in Iran, namely the Health Transformation Plan (HTP). | descriptive analytical study | WHO method | NO | Secondary data | Original Article/ English | 2014 and 2015 | Iranian households |
| 49 | Kazemi-Karyani, Ali et al. 2020 ([71](#_ENREF_71)) | Estimate socioeconomic inequality differences in CHE between urban and rural areas of Iran after the implementation of the HTP during 2017. | Representative survey | WHO method, Wagstaff’s normalized concentration index | Yes | Secondary data | Original Article/ English | 2017 | Iranian households |
| Determinants of exposure to CHE: Socioeconomic status (SES) (+), outpatient services (+), health insurance coverage (-).Sex(Female headed)(+), households with an elderly person(+), with no under-5-year-old children(+), with a family size of 1–2 people (+), living in rural areas (+), with individuals having chronic specific diseases (+), and those who utilize inpatient (+) and outpatient (+), region of residency (+). | | | | | | | | | |
| 50 | Kavosi, Z et al. 2020 ([72](#_ENREF_72)) | Identify and explain the interactions and network of the relationship between influential factors of out-of-pocket payments for health services. | futures study | futures study and cross-impact analysis | NO | Square-matrix questionnaire | Original Article/ English | 2015 | Iranian household |
| Determinants of exposure to CHE: context factors: Health sector inflation(+), Exchange rate(+), the aging population and life expectancy increase(+), disease pattern change(+), disease outbreaks(+), clinical guidelines (complication, application for monitoring and establishing basic benefits package)(-), the tariff growth rate(+), population coverage/ Risk factors: service coverage(-), cost coverage(-)/ Target factors: referral path system(-), the use of expensive services(+), under the table fees and informal payments(+), induced demand due to information asymmetry between patient and doctor(+)/ Supplementary insurance(-), quality of health care(+), private sector outpatient services consumption(+), use of outpatient services of the public sector (public-armed-charity)(+), private inpatient services consumption(+), use of public inpatient services(+), access to health services(+), privatization and amount of private sector activity development(+), rational prescription of medicines and services(-), medicine prices (internal-external)(+)/ Discrete factors: monitoring and ensuring the implementation of policies(-), the willingness of people to use special service or the taste of people(+), medical education policies(+), health services tariff (process and base of tariffs)(+), state social support organizations/ Second lever and discrete factors: life style and self-care behavior(+), different tariff of private and public sectors(+), aggregation of insurance institutions(-), the allocation of resources to the health sector (budgeting)(+), increase in ran population(+), high tariff of dental services(+). | | | | | | | | | |
| 51 | Hajibabaei, Hamidreza et al. 2020 ([73](#_ENREF_73)) | Estimate the health expenditures function and find out the determinants of health expenditures | Analytical research | Panel data method | NO | Secondary data | Original Article/ English | 2010 until 2014 | Developing countries and Iranian household |
| Determinants of exposure to CHE: Income (GGHE-D as percentage of GDP) (+), The demographic growth the proportion of the young (e.g. under 15 years old) and old people (e.g. above 65 or 75 years old) (+), The technological progress (+), the role of real prices in determining the demand for health care (+), the medical density (It is defined by physicians as per thousand population) (+), and used to account for the supply of healthcare (+), Institutional factors (Two approaches are used. The first distinguishes the effects of instructions on remuneration. The second distinguishes the effects of the type of national health system (e.g. contractual system or integrated system), Social characteristics: distribution of income, distribution of education, skills, jobs, opportunities, and expectations for the future(+), Education(-). Determinants of exposure to CHE: Socioeconomic status (SES) (+), live in rural and urban (+), outpatient services (+), health insurance coverage (-).Sex(Female headed)(+), households with an elderly person(+), with no under-5-year-old children(+), with a family size of 1–2 people (+), living in rural areas (+), with individuals having chronic specific diseases (+), and those who utilize inpatient (+) and outpatient (+), region of residency (+). | | | | | | | | | |
| 52 | Yahyavi Dizaj, Jafar et al. 2020 ([74](#_ENREF_74)) | Evaluate the effect of the presence and age of elderly members on health care costs of the households in Iran. | secondary analysis | Linear regression analysis | No | Secondary data | Journal Article- Persian | 2016 | Elderly people over 65 years old in Iran |
| Determinants of exposure to CHE: having a smoker member(+), having an income-earner member(-), living in urban areas(+), household head education(-), health development rate of the province of residence(+), Elderly population(+). | | | | | | | | | |
| 53 | Hsu, Justine. 2020 ([75](#_ENREF_75)) | Protect the Iranian population from the consequences of catastrophic and impoverishing OOP payments and to ensure more equitable financing of the health system. | descriptive analytical report | Report | No | Secondary data | Original Article/ English | 2007-2015 | Iranian households |
| Determinants of exposure to CHE: year, area of residence of the household (i.e. rural or urban), characteristics of the household head (i.e. sex, insurance status, educational level, employment status, literacy, civil status), whether at least one household member was younger than 5 years and whether at least one household member was older than 60 years. the household is in a rural or an urban area and the employment status of the household head. employment status, the composition of the household also influences the probability of catastrophe (Those with an elderly member over the age of 60 years or with a child under 5 years of age), the level of education of the head of the household, households in which the head is a female or a student. | | | | | | | | | |
| 54 | Woldemichael, A et al. 2021 ([76](#_ENREF_76)) | Analyses impact of OOP payments for dental services on prevalence CHE among Iranian households during 2018. | cross-sectional analysis | WHO method | Yes | United Nations designed and approved questionnaire | Original Article/ English | 2018 | Iranian households |
| Determinants of exposure to CHE: Demographic variables: Sex of household head (Female+), Age of household head (≥ 66 +), Household had ≤5-year-old child (No+), Household had ≥66-year-old member (Yes+), Socioeconomic variables: Educational status of household head (illiterate+), Wealth index of households (Poorest+) , Insurance coverage (Yes+) , Ecological variables: Geographical location of household (Rural +), HDI*of province (High +) | | | | | | | | | |
| 55 | Moradi, G et al. 2021 ([77](#_ENREF_77)) | Investigate the percentage of households with disabled children aged 0 to 8 years who had faced CHE due to the health costs of these children in Iran. | cross-sectional study | WHO method | Yes | WHO questionnaire | Original Article/ English | 2020 | Households with disabled children aged 0 to 8 years in five provinces in Iran |
| Determinants of exposure to CHE: Head of household being female(+), poor economic status of the household(+), lack of supplementary insurance by a child with disabilities(+), having a child with mental disability(+), and type of basic health insurance((having Iranian Health Insurance+). | | | | | | | | | |
| **The data extraction and incidence and intensity of CHE, % (Provinces population level)** | | | | | | | | | |
| 56 | Karami, M et al. 2009 ([78](#_ENREF_78)) | Describe the magnitude and distribution of CHE in Kermanshah western Iran. | descriptive study | WHO method | NO | WHO questionnaire | Original Article/ English | 2008 | Kermanshah |
| Determinants of exposure to CHE: have a family member younger than 12 year old or older than 60 year old(+), families have a member suffering from chronic condition(+), families headed with old people(+), females and those with disabilities(+), the unemployed or poor people(+), and those with reduced access to health insurance(+), Insurance coverage and complementary insurance coverage status(-). | | | | | | | | | |
| 57 | Kavousi,z et al. 2009 ([79](#_ENREF_79)) | Quantify and compare the proportion of households facing CHE in years 2003 compared to 2008, and to identify the factors that contributed to these expenditures. | Longitudinal study | WHO method Chi-square test. | NO | WHO questionnaire | Journal Article- Persian | 2003-2008 | Tehran |
| Determinants of exposure to CHE: use of expensive inpatient care(+), use of essential dental care (not covered in insurance packages)(+), number of outpatient services(+), having member over 65y(+), having disabled member(+), and lower economic status(+) | | | | | | | | | |
| 58 | Moghimi, M et al. 2009 ([80](#_ENREF_80)) | Exploring the performance of Government Rule in Supporting and Decreasing CHE of Cancer Patients in Zanjan Province in 2007-2008 | descriptive-analytic cross-sectional study | WHO method | NO | Self-administered questionnaire. | Journal Article- Persian | 2007-2008 | Zanjan |
| Determinants of exposure to CHE: household income status(-), household size(+), disability of the head of the family due to illness(+), wife, or child(+), rented house(+), insufficient coverage of supplementary insurance(+), lack of access to all medical services in the city(+), village and the need to continue treatment in large cities(+), use of private diagnostic services(+), lack of coverage expensive drug by insurance organizations(+), diagnosis of the disease in advanced stages(+) | | | | | | | | | |
| 59 | Ghiasvand, H/2010 ([81](#_ENREF_81)) | Identify factors that influence CHE in patients of teaching hospitals affiliated to Iran University of Medical Science in 2009. | cross-sectional study | Data analyzes | NO | Self-administered questionnaire. | Journal Article- Persian | 2008-2009 | Tehran |
| Determinants of exposure to CHE: gender of the household’s head (Female+), health status of the member of household (+), the size of household, residency in Tehran city(+), number of previous hospitalization(+), having a house(-), the level of income(-), and finally complementary health insurance coverage(-). | | | | | | | | | |
| 60 | Daneshkohan, A et al. 2011 ([82](#_ENREF_82)) | Estimate FFCI and quantify extent of household CHE | A cross-sectional study | WHO method | No | Self-administered questionnaire. | Original Article/ English | 2008 | Kermanshah |
| Determinants of exposure to CHE: retrospective payment mechanisms(+), especially fee-for-service(+), Insurance Coverage(-), member with chronic condition(+), member older than 60 years old(+), member younger than 12 year old(+), Gender of the head of the household(women+) | | | | | | | | | |
| 61 | Ghiasvand, Hesam et al. 2011 ([83](#_ENREF_83)) | Assessed the performance of Iranian health insurance schemes in protection the patients against catastrophic medical payment. | cross sectional analytical study | Logit regression model | Yes | self-administered questionnaire. | Journal Article- Persian | 2009 | Tehran |
| Determinants of exposure to CHE: Household’s head gender(women-), Number of hospitalization(+), Residency in Tehran(-), Income level(-), other family member’s illness(+), Ownership of house(-), Number of household’s members(+), the coverage of complementary health insurance(-) | | | | | | | | | |
| 62 | Kavosi, Z et al. 2012 ([84](#_ENREF_84)) | Assessed change in household CHE and inequality in facing such expenditures in south-west Tehran | longitudinal study | WHO method | Yes | WHO questionnaire | Original Article/ English | 2003-2008 | Tehran |
| Determinants of exposure to CHE: health care utilization(+) and health care insurance status(-), Socioeconomic status(+), utilization of dentistry(+), and outpatient services(+), general inflation in the country(+) and in part because of increasing health care tariffs(+), increasing consumption of expensive high-tech health care services(+), member over 65years(+), having disabled members(+). | | | | | | | | | |
| 63 | Amery, H et al. 2013 ([85](#_ENREF_85)) | Measure the catastrophic expenditures of health services and effective indicators | cross sectional | WHO method | Yes | WHO questionnaire | Journal Article- Persian | 2012 | Torbat Heydarieh |
| Determinants of exposure to CHE: Under the age 5 in the family(+), the existence of a 65 or older family member(+), family size(+), The use of medicines, and diagnostic tests(+). | | | | | | | | | |
| 64 | Amery, H et al. 2013 ([86](#_ENREF_86)) | Examine the CHE and its influential factors. | cross sectional | WHO method | Yes | WHO questionnaire | Journal Article- Persian | 2011 | Yazd |
| Determinants of exposure to CHE: The use of medicine, diagnosis, and inpatient services (+), members below 5 years old in household (+) and family size (+). | | | | | | | | | |
| 65 | Asefzadeh, Saeed et al 2013 ([87](#_ENREF_87)) | Calculate households encountered with CHE in Qazvin, Iran. | descriptive-analytic cross-sectional study | WHO method | NO | Self-administered questionnaire. | Journal Article- Persian | 2011 | Qazvin |
| Determinants of exposure to CHE: Household economic status(-), frequency of use of outpatient services(+), inpatient services(+), and Out-of-Pocket (OOP) payment for medicine, laboratory, dentistry, radiology, Physiotherapy and rehabilitation(+) | | | | | | | | | |
| 66 | Nekoeimoghadam, Mahmood et al. 2013 ([88](#_ENREF_88)) | Measure CHE in Kerman province, Iran, and the affecting factors. | descriptive-analytical retrospective research | WHO method, chi-square test and logistic regression | Yes | Secondary data | Journal Article- Persian | 2008 | Kerman |
| Determinants of exposure to CHE: health services utilization(+), particularly inpatient(+), outpatient and dental care services(+), radiology, sonography, radiotherapy, echocardiography, MRI, exercise test(+). | | | | | | | | | |
| 67 | Rezapour, Aziz et al. 2013 ([89](#_ENREF_89)) | Determine the effects of OOP for health care services on households in Tehran (2013) | cross-sectional study | WHO method | Yes | WHO questionnaire | Original Article/ English | 2013 | Tehran |
| Determinants of exposure to CHE: education status of household head(-), household size(+), and number of the times that outpatient health services(+), preschool children living in HHs(+), member with chronic illness(+). | | | | | | | | | |
| 68 | Sadeghiyeh Ahari, Saeid et al. 2013 ([90](#_ENREF_90)) | Exposure rate of the catastrophic health care costs in patients with ESRD in Buali Hospital dialysis department. | descriptive-analytic cross-sectional study | WHO method | NO | WHO questionnaire | Journal Article- Persian | 2013 | Ardabil |
| Determinants of exposure to CHE: The place of residence(+), presence of side income in other members of family(-), going on vacation(-). | | | | | | | | | |
| 69 | Yavangi, M. et al. 2013 ([91](#_ENREF_91)) | Determine the total expenditure and OOP on pregnancy complications in Tehran, the capital of Iran. | cross-sectional study | WHO method | NO | Self-administered questionnaire. | Original Article/ English | 2009 | Tehran |
| 70 | Anbari, Z et al. 2014 ([92](#_ENREF_92)) | Evaluating some health expenditure of inpatient and outpatient care as well as assessing the predictors of catastrophic costs for inpatient care in one of central provinces of Iran. | cross-sectional | WHO method | Yes | standard questionnaire in health utilization care | Original Article/ English | 2014 | Markazi |
| Determinants of exposure to CHE: age range 40-59 years(+), and being in the lower levels of wealth index (+). | | | | | | | | | |
| 71 | Ghafoori, Mohammad Hossein et al. 2014 ([93](#_ENREF_93)) | Determine disparities in health expenditures by means of different approaches. FFC, CI in health expenditure, Lorenz curve. | A cross-sectional population based study | WHO method | No | WHO questionnaire | Original Article/ English | 2012 | Tehran |
| 72 | Ghiasvand, Hesam et al. 2014 ([94](#_ENREF_94)) | Calculate the proportion of hospitalized patients exposed to CHE, its determinant factors and its distribution. | cross sectional health survey | WHO method | Yes | Self-administered questionnaire. | Original Article/ English | 2012 | Tehran |
| Determinants of exposure to CHE: The weakness of economic status of households(+), the not well designed prepayments schemes(+), the educational level of the patient's family head(-), the sex of the patient's family head(male-), hospitalization day numbers(+), having made any out of hospital payments linked with the same admission(+) and households annual income levels(-) | | | | | | | | | |
| 73 | Ghoddoosinejad, Javad et al. 2014 ([95](#_ENREF_95)) | Calculate households encountered with CHE in Ferdows, Iran. | descriptive-analytic cross-sectional study | WHO method | No | Self-administered questionnaire. | Original Article/ English | 2014 | Ferdows |
| Determinants of exposure to CHE: Use of dentistry services and hospital care(+). | | | | | | | | | |
| 74 | Kavosi, Zahra et al. 2014 ([96](#_ENREF_96)) | Investigated the Household Financial Contributions (HFCs) to the health system. | cross-sectional descriptive study | WHO method | Yes | WHO questionnaire | Original Article/ English | 2012 | Shiraz |
| Determinants of exposure to CHE: household economic status(-), the basic and supplementary insurance status of the head of the household(-), existence of individuals in the household who require chronic medical care(+), use of dental and hospital care(+), rural location of residences(+), frequency of use of outpatient services(+), and Out-of-Pocket (OOP) payment for physician visits(+), Existence of persons over age 65(+), Existence of persons under age 5(+), Percentage of household capacity to pay devoted to healthcare service(+). | | | | | | | | | |
| 75 | Kavosi, Z et al. 2014 ([97](#_ENREF_97)) | Determine the percentage of households with cancer patients that face CHE. | descriptive-analytic cross-sectional study | WHO method | NO | WHO questionnaire | Original Article/ English | 2011 | Shiraz |
| Determinants of exposure to CHE: Insurance status (-), Type of insurance (-), residence (+), use of outpatient services (+), type of treatment and other family members who refrained from using healthcare services (+), Age of head of household (+), Economic status (+), Family size (+), Type of cancer (+), Type of treatment (+), Refraining from using healthcare services (+). | | | | | | | | | |
| 76 | Asma Sabermahani et al. 2014 ([98](#_ENREF_98)) | Investigate factors affecting the probability of CHE exposure among households in Tehran. | Retrospective descriptive study | WHO method | Yes | Secondary data | Journal Article- Persian | 2011 | Tehran |
| Determinants of exposure to CHE: Households with the number of members under 5 years or over 65 years (+), Unemployed or less educated head (+), Households with a chronic patient (+), Households without insurance supports (+), Percapita household expenditure (+) Number of the employed person in household(-). | | | | | | | | | |
| 77 | Panahi, Hossein et al. 2014 ([99](#_ENREF_99)) | Identify factors that influence CHE by patients in of Tabriz, Iran. | A descriptive analytical study | WHO method | NO | Self-administered questionnaire. | Journal Article- Persian | 2011-2012. | Tabriz |
| Determinants of exposure to CHE: age of members(elderly people/ children)(+), and gender (female patients(+), person with chronic diseases(+), admission to a private hospital and lower household wealth | | | | | | | | | |
| 78 | Khammarnia, M et al. 2014 ([100](#_ENREF_100)) | Investigate the households' impoverishment due to the healthcare costs in Shiraz in 2012. | cross sectional study | WHO method | Yes | WHO questionnaire | Original Article/ English | 2012 | Shiraz |
| Determinants of exposure to CHE: household's economic status(+), place of living(+), and consumption of outpatient services(+). | | | | | | | | | |
| 79 | Hatam, Nahid et al. 2015 ([101](#_ENREF_101)) | Identify the determinants of exposure to CHE in the hospitalized patients, in the selected hospitals of SUMS, Iran. | descriptive-analytic cross-sectional study | WHO method, chi-square test, T-test, Mann-Whitney, Logistic regression | Yes | WHO questionnaire | Original Article/ English | 2013 | Shiraz |
| Determinants of exposure to CHE: Household economic status (-), Type of hospital (+), Ward (+), Household head’s state of health insurance (-), Patient’s supplementary insurance status (-), Household head’s complementary insurance status (-), Household size (+), Gender of patient(female+), Gender of household head(female+), Person under 5 years old (+), Person over 65 years old (+), Time of diagnosis (+), Hospitalization duration (+), Ability to pay (-). | | | | | | | | | |
| 80 | Tofighi, Shahram et al. 2015 ([102](#_ENREF_102)) | Calculation of catastrophic costs were extracted from both of old and non- old groups | cross-sectional study | WHO method | NO | WHO questionnaire | Original Article/ English | 2011 | Tehran |
| Determinants of exposure to CHE: over 60 years of age (aging)(+). | | | | | | | | | |
| 81 | Davari, Majid et al. 2015 ([103](#_ENREF_103)) | Determining and comparing socioeconomic status (SES) among different periods, and made an attempt to evaluate households’ health financial protection in different quintiles after implementation of Family Physician (FP) program. | A time trend study | WHO method | NO | Secondary data | Original Article/ English | 2004 and 2011 | Chaharmahal and Bakhtiary |
| Determinants of exposure to CHE: Hospitalization(+), quintiles status(+), education(-), income(-), occupation(-), home status(-) and family size(+), rural areas(-), unemployment rate(+), insurance coverage(+), utilization rate of inpatient and outpatient services(+). | | | | | | | | | |
| 82 | Fattahi, Shahram et al. 2015 ([104](#_ENREF_104)) | Identified the factors affecting the cost of misery burden of health to be able to reduce these costs and can be effective step to identify vulnerable groups. | cross-sectional study- case study | Data collection and data analyzes | NO | self-administered questionnaire. | Journal Article- Persian | 2012-2013 | Hossein Abad District of Uremia |
| Determinants of exposure to CHE: wealth index(-), gender of household head(female+), household size(+), presence of household members less than 12 years(+), job status of household head(-), and number of hospital services to be covered by compulsory insurance and supplemental insurance(-). | | | | | | | | | |
| 83 | Bagheri faradonb, S et al. 2016 ([105](#_ENREF_105)) | Investigate the catastrophic and impoverishing health expenditure in Tehran urban population. | cross-sectional study | WHO method | Yes | WHO questionnaire | Journal Article- Persian | 2013 | Tehran |
| Determinants of exposure to CHE: Head of household education level(-), the presence of people over 60 years in household(+), the use of inpatient services and the volume of use(+), informal payment(+). | | | | | | | | | |
| 84 | Ghiasi, A et al. 2016 ([106](#_ENREF_106)) | Investigates the CHE and its determinants among the household residents of Zabol. | cross sectional study | WHO method | Yes | WHO questionnaire | Journal Article- Persian | 2013-2014 | Zabol |
| Determinants of exposure to CHE: Education of the head of the household (-), medical expenditure(+), pharmaceutical expenses(+). | | | | | | | | | |
| 85 | Juyani, Yaser et al. 2016 ([107](#_ENREF_107)) | Investigate on what extent Multiple sclerosis patients face catastrophic costs. | descriptive-analytic cross-sectional study | WHO method | Yes | Self-administered questionnaire. | Original Article/ English | 2014 | Ahvaz |
| Determinants of exposure to CHE: Brand of drug(+), housing, income(-), health insurance(-), hospitalization(+), doctor visit(+), rehabilitation services(+). | | | | | | | | | |
| 86 | Piroozi, B et al. 2016 ([108](#_ENREF_108)) | Explore the percentage of households facing CHE after the implementation of HSEP and the factors that determine CHE. | descriptive-analytic cross-sectional study | WHO method | Yes | WHO questionnaire | Original Article/ English | 2015 | Sanandaj |
| Determinants of exposure to CHE: household economic status(-), presence of elderly or disabled members in the household(+), Household having member(s) under 5 years old(+), utilization of inpatient, rehabilitation services(+), dental care(+), Status of the basic health insurance(-), status of supplementary health insurance(-), gender of the household head(male-), Household size(+). | | | | | | | | | |
| 87 | Rezapour, A et al. 2016 ([109](#_ENREF_109)) | determine the equity in health care payments and determining factors among households in Hamedan | cross-sectional study | WHO method | Yes | WHO questionnaire | Original Article/ English | 2014 | Hamedan |
| Determinants of exposure to CHE: having members under 6 years or over 60 years in household(+), household size(+), household head gender(female+), employment of household head(-), households' income quintile(+), existence of the disabled member in households(+), and the education level of the household’s head(-). | | | | | | | | | |
| 88 | Rezapour, A et al. 2016 ([110](#_ENREF_110)) | Estimate the odd-ratio of factors affecting families' exposure to catastrophic and impoverishing health expenditures | cross-section study | WHO method | Yes | WHO questionnaire | Journal Article- Persian | 2013 | Tehran |
| Determinants of exposure to CHE: The presence of people over 60 years in households (+), the use of inpatient services and the volume of use (+), Informal payments (+), insurance coverage (+), insurance coverage status (+). | | | | | | | | | |
| 89 | Khadivi, Reza et al. 2016 ([111](#_ENREF_111)) | Determine the utilization rate of health services among construction workers and their families. | Descriptive analytical study | WHO method | NO | WHO questionnaire | Journal Article- Persian | 2013 | Isfahan |
| Determinants of exposure to CHE: Hospitalization (+) | | | | | | | | | |
| 90 | Almasi, Mojtaba et al. 2016 ([112](#_ENREF_112)) | Factors affecting the crippling cost of dialysis patients | descriptive-analytic cross-sectional study | A Probit model | NO | WHO questionnaire | Journal Article- Persian | 2014 | Urmia |
| Determinants of exposure to CHE: wealth index(-), gender of household head(male+), place of residence(rural+), presence of members in need of care(+), job status of household head(-), number of dialysis services to be covered by compulsory insurance and supplemental insurance(-), number of dialysis services(+), presence of members in need of care(+). | | | | | | | | | |
| 91 | Homaie Rad, E et al. 2017 ([113](#_ENREF_113)) | Evaluated OOP for outpatient, inpatient, and drug services, and CHE using a before-and-after the reform analysis | cross-sectional research Before and after analysis | WHO method | NO | Secondary data | Original Article/ English | 2013 and 2015 | Guilan |
| Determinants of exposure to CHE: Family income(-), the presence of children under 5 years of age(+), members of the family more than 70 years old(+), the number of illiterate people in the family(+), and the number of women in the family(+). | | | | | | | | | |
| 92 | Moradi, G et al. 2017 ([114](#_ENREF_114))] | Exploring the likelihood of facing CHE among households with members suffering from dialysis, kidney transplant, or (MS) after the implementation of HSEP. | descriptive-analytical study | WHO method | Yes | WHO questionnaire | Original Article/ English | 2015 | Kurdistan |
| Determinants of exposure to CHE: patient's education(-), household income(-), patient supplementary insurance status(-), type of special disease(+) a family member with a special disease(+) patient residence(rural+), frequency of using inpatient services(+) use of dental care(+) and use of rehabilitation services(+). | | | | | | | | | |
| 93 | Rezapour, A et al. 2017 ([115](#_ENREF_115)) | Analyze CHE among households with and without chronic NCDs in Hamedan. | descriptive-analytic cross-sectional study | WHO method | Yes | WHO questionnaire | Original Article/ English | 2011 | Hamedan |
| Determinants of exposure to CHE: Lower economic status(+), lower household size(+), and high utilization of health care(+), households with chronic NCDs(+) | | | | | | | | | |
| 94 | Mobaraki, Hosein et al. 2018 ([116](#_ENREF_116)) | Determine the percentage and characteristics of older adults facing with the CHE in Tehran, Iran. | cross-sectional study | WHO method | Yes | WHO questionnaire | Original Article/ English | 2017 | Tehran |
| Determinants of exposure to CHE: Household income quintile(-), Home ownership(-), employment status(-), household size(+), Disabled family member(+), and supplementary insurance(-). | | | | | | | | | |
| 95 | Khammarnia, Mohammad et al. 2018 ([117](#_ENREF_117)) | Evaluate the effectiveness of the health transformation plan, this study was conducted with the goal of determining the health expenditures by households after implementation of this new plan. | cross sectional- descriptive study | WHO method | NO | WHO questionnaire | Journal Article- Persian | 2015 and 2016 | Zahedan |
| Determinants of exposure to CHE: Drug fees(+), use physiotherapy services(+), outpatient services(+), having a family member who needs to be taken care of at home(+), and a family member who needs to be taken care of at a hospital(+), Lack of access to healthcare services(+), high dispersion of the population(+), insurance status(-), income status(-). | | | | | | | | | |
| 96 | Khammarnia, M.et al. 2018 ([118](#_ENREF_118)) | Examine the households’ impoverishment due to health expenditure after HTP. | cross-sectional study | WHO method | Yes | WHO questionnaire | Original Article/ English | 2017 | Sistan and Baluchestan |
| Determinants of exposure to CHE: Living in a rural area(+), unemployment(+), economic status(-),inpatients and outpatient costs(+), having supplementary insurance(-) | | | | | | | | | |
| 97 | Motlagh, S. N. et al. 2018 ([119](#_ENREF_119)) | To measure the fairness of health care financing and to identify incidence rate of CHE and its most important determinants before and after implementing the HSEP among households in one province of Iran (Lorestan). | cross-sectional research | WHO method | Yes | SCI Questionnaire | Original Article/ English | 4/2012-3/2015 | Lorestan |
| Determinants of exposure to CHE: Economic status of households(-), location of households (urban or rural+), number of people over the age of 65 and under the age of 5 in the household(+), age and sex(female) of household head(+), insurance status of households(-). | | | | | | | | | |
| 98 | Piroozi, B et al. 2019 ([120](#_ENREF_120)) | Measure the proportion of households facing CHE and identifying the effective factors on household’s exposure to CHE. | cross-sectional study | WHO method | Yes | WHO questionnaire | Original Article/ English | 2018 | Kurdistan |
| Determinants of exposure to CHE: low socio-economic status (+), supplementary health insurance (-). | | | | | | | | | |
| 99 | Mehdizadeh, P et al. 2019 ([121](#_ENREF_121)) | Analyzed exposure to CHE and factors affecting them among the health staffs affiliated to army medical universities in Tehran | descriptive-analytic cross-sectional study | WHO method | Yes | WHO questionnaire | Original Article/ English | 2016 | Tehran |
| Determinants of exposure to CHE: used dental services (+), households with 3 members and less(+), households with lower education level(+), households with two or more outpatient visits(+). | | | | | | | | | |
| 100 | Rezaei, Satar et al. 2019 ([122](#_ENREF_122)) | Measure and decompose socioeconomic inequality in CHE among households in Kermanshah province, Western of Iran. | cross-sectional study | WHO method | NO | Secondary data | Original Article/ English | 2017 | Kermanshah |
| Determinants of exposure to CHE: socioeconomic status(-), health insurance coverage(-). | | | | | | | | | |
| 101 | Kazemi-Galougahi, M. H et al. 2019 ([123](#_ENREF_123)) | Analyze CHE trend over time and to determine its determinants. | descriptive study | WHO method | Yes | WHO questionnaire | Original Article/ English | 2003, 2008 and2015 | Tehran |
| Determinants of exposure to CHE: Lack of Insurance(+), economic status(-), Female household head(+), Having member ≥65 in Household(+), Having member ≤5 in Household(+), Household size(+), Having disabled member in household(+), Dentistry service usage(+), Inpatient service usage(+), Outpatient service usage(+), Inflation(+), the implementation of the Iranian targeted subsidy plan(+). | | | | | | | | | |
| 102 | Barfar, Eshagh. et al. 2019 ([124](#_ENREF_124)) | Measure CHE for households with severe mental disorders (SMDs) patients. | cross-sectional study | WHO method, Logistic regression, concentration index, Decomposition analysis | NO | World Health Survey questionnaire | Original Article/ English | July 2017 to March 2018 | Tehran |
| Determinants of exposure to CHE: the age range of 40 to 59-years-old for the household heads(-), a rising education level of the household head(-), utilization of dental(+), rehabilitation(+), and medication services(+), Households in the higher economic quintile(-),increasing the households' monthly expenditure(-). | | | | | | | | | |
| 103 | Nemati, Esmat et al. 2020 ([125](#_ENREF_125)) | Investigating the OOP and exposure of households with CHE following the implementation of a health transformation plan in Tabriz, Iran. | descriptive-analytic cross-sectional study | WHO method | Yes | WHO questionnaire | Original Article/ English | 2017 | Tabriz |
| Determinants of exposure to CHE:Gender(female), Age(+), Marital status(+), Education(-), Employment status(-), Covered by insurance(-), Income(-), Size of the household(+), Dentist services(+), Pharmaceutical services(+), Radiology services(+), Physiology services(+), The presence of people under care(+), Marital status(+). | | | | | | | | | |
| 104 | Piroozi Bakhtiar et al. 2020 ([126](#_ENREF_126)) | Assess the prevalence and intensity of CHE relating to type 2 diabetes mellitus care and inequality in facing such expenditures in Iran. | cross-sectional study | WHO method | Yes | Self-administered questionnaire. | Original Article/ English | 2019 | Isfahan, Sanandaj,Sabzevar |
| Determinants of exposure to CHE: Socioeconomic status(-), being female(+), older age(+), education(-), marital status(+), employment status(-), use of inpatient services(+), household size(+), household assets(-), living place(rural+), type of health insurance(-), duration of diabetes(+), and the complications of diabetes(+) | | | | | | | | | |
| 105 | Ahmadi, Razieh et al. 2020 ([127](#_ENREF_127)) | Calculate the percentage of CHE after implementing the plan and compare that with CHE before the plan at the same households. | descriptive-analytic cross-sectional study | WHO methodology | Yes | WHO questionnaire | Original Article/ English | 2020 | Yazd city |
| Determinants of exposure to CHE: household size(+), member ≥65 years in household(+), the economic status(-), dental services(+), and using inpatients services(+). | | | | | | | | | |
| 106 | Dabbaghi, F. et al. 2020 ([128](#_ENREF_128)) | Determine the burden of CHCs on patients admitted to selected hospitals in Semnan and Shahrood. | Descriptive-analytic study | WHO methodology | Yes | Researcher-made questionnaire | Original Article/ English | 2017 | Semnan and Shahrood |
| Determinants of exposure to CHE: Type of illness or disability of family members(+), Presence of people aged > 65 years or < 5 years in the household(+), Household income level(-), Household head’s gender(female-), Number of hospitalization(+), Household supplementary insurance coverage(-), Number of household members(+), Basic insurance coverage of household members(-), Type of household head’s occupation(-). | | | | | | | | | |
| 107 | Khammarnia, M.et al. 2020 ([129](#_ENREF_129)) | Measure the household CHE and FFCI in Sistan-Baluchistan Province after the implementation of HTP. | cross-sectional study | WHO methodology | Yes | WHO questionnaire | Original Article/ English | 2017 | Sistan-Baluchistan |
| Determinants of exposure to CHE: place of residence(+), having members aged more than 65 years(+), having members with disabilities and in need of care(+), the use of health services(+),the use of dental(+), rehabilitation(+), diagnostic and laboratory(+), and inpatient services(+). | | | | | | | | | |
| 108 | Vahedi, S et al. 2020 ([130](#_ENREF_130)) | Explain the contributors of the unequally distributed among disadvantaged populations in Hamadan, Iran. | descriptive-analytic study | WHO methodology | Yes | WHO questionnaire | Original Article/ English | 2014 | Hamedan |
| Determinants of exposure to CHE: poor economic status(+), lower household size(-), lack of supplementary insurance(+), and the number Masoudi hospitalizations(+) | | | | | | | | | |
| 109 | Ahmadi, F et al. 2021 ([131](#_ENREF_131)) | Calculated OOP, CHE, and impoverishing health spending attributed to breast cancer in Iran. | cross-sectional household study | WHO methodology | Yes | WHO questionnaire | Original Article/ English | 2019 | Urmia |
| Determinants of exposure to CHE: Place of living (+), Household dimension (+), Age(+), Having insurance(-), Education level(-), Marital status(-). | | | | | | | | | |
| 110 | Sabermahani, A et al. 2021 ([132](#_ENREF_132)) | Analyze all aspects of OOP, especially after the Health Transformation Plan in Iran | cross-sectional study | Interview | No | self-administered questionnaire | Original Article/ English | October 2017 to March 2018 | Kerman |
| Determinants of exposure to CHE: length of stay in hospitals(+), the need for the presence of next of kin(+), and provision of healthcare services out of hospitals(+). | | | | | | | | | |
| 111 | Ravangard Ramin et al. 2021 ([133](#_ENREF_133)) | Measure the percentage of households facing CHEs and the factors associated with the occurrence of CHEs in Shiraz, Iran in 2018. | cross-sectional study | WHO methodology | Yes | WHO questionnaire | Original Article/ English | 2018 | Shiraz |
| Determinants of exposure to CHE: households living in rented houses(+), households with disabled members(+), households with children under 5 years old(+), those without supplementary health insurance coverage(+). | | | | | | | | | |
| 112 | Farid Gharibi et al. 2021 ([134](#_ENREF_134)) | Evaluate multiple sclerosis (MS) treatment costs and the resulting economic impact imposed on MS patients in Iran. | cross-sectional study | WHO methodology | No | self-administered questionnaire | Original Article/ English | 2018 | the East Azerbaijan province |
| Determinants of exposure to CHE: medication (+), rehabilitation care (+), and physician visits (+), Type of basic insurance (+), Resident (in Tabriz+), Age of patient at disease incidence (+), Duration of disease (years) (+). | | | | | | | | | |

### **Supplementary 9. Factors associated with catastrophic health expenditure**

| **Category** | **Criteria** |
| --- | --- |
| Socioeconomics characteristics of HH | Household economic status (Q1 vs. Q5) |
|  | Place of residence (urban, rural, remote areas) |
|  | Health insurance status of HH |
|  | Supplementary insurance status of HH (head and members) |
|  | Wealth index (income deciles) |
|  | Homeownership |
|  | The type of health insurance |
|  | Per capita household or housing infrastructure |
|  | Household total expenditure or Per capita household expenditure |
|  | Number of the Insured / Uninsured in the Informal Sector |
|  | Insurance expenditure |
| Demographic characters of Household | Household size |
|  | Gender of household head (HHH) |
|  | Education level of HHH |
|  | Employment status of the HHH or members |
|  | Education level of household members or patient |
|  | The age of HHH |
|  | Marital status of HHH |
|  | Gender of patients |
|  | Male ratio of household |
|  | Households in which the head is a student |
|  | Age of patient at disease incidence |
| Vulnerable person in HH | Having elderly member (over 60 years of age) in HH |
|  | Have under 5y children in HH |
|  | Having member with chronic disease and NCDs (In particular; cancer, renal dialysis, MS, SMDs, diabetic) |
|  | Having member with disability in HH |
|  | Under 12 y member living in Household |
|  | Having member in HH in need of care |
|  | Health status of the member of household |
|  | Having a smoker member |
| Health care utilization by HH members | Using inpatient services and the volume of use by HH members and length of stay |
|  | Using outpatient services and the volume of use by HH members |
|  | Using dentistry services by HH members |
|  | Using medicines and equipment |
|  | Using diagnostic services (clinical services of Pathology and Laboratory Medicine, Radiology, sonography, radiotherapy, echocardiography, MRI, exercise test, and Nuclear Medicine) |
|  | Using Physiotherapy and rehabilitation service |
|  | Using private services by HH members |
|  | Health services utilization |
|  | Utilizing cancer treatments |
|  | Utilizing dialysis services and the volume of use by HH members |
|  | Utilizing ambulatory |
|  | Use of drug addiction cessation services |
| Health Expenditure Indicators | Basic health insurance coverage ratio |
|  | Complementary health insurance coverage status |
|  | Inequality in access (financial, geographical, and cultural) to healthcare services and safe water |
|  | The medical density (It is defined by physicians as per thousand population and other educated health workers.) |
|  | Informal payments or under-the-counter payment |
|  | Distribution of income, education, skills, jobs, opportunities, physician, specialized manpower, health expenditures, and expectations |
|  | Household health expenditures |
|  | Increasing consumption of expensive high-tech health care services |
|  | Health care tariff growth rate |
|  | Physician visits |
|  | Change of consumption towards branded drugs |
|  | Time of diagnosis |
|  | Refraining from using healthcare services |
|  | High inflation rates in the health sector |
|  | Households’ Willingness to Pay for Health Services |
|  | Lifestyle pattern and self-care behavior |
|  | Payment mechanisms |
|  | Adoption of public insurance law |
|  | The implementation of health transformation plan in 2014 |
|  | Per capita public health costs |
|  | Quality of health care |
|  | Type of hospital |
|  | Induced demand (consumer or supplier) |
|  | Weakness in service delivery and surveillance system |
|  | Real prices of health services |
|  | Reduction of accumulation of insurance resources |
|  | Multiplication of basic insurance funds |
|  | Absence of clinical guidelines |
|  | Disease outbreaks and pandemics |
|  | Lack of financial protection |
|  | Out-of-pocket Share in Total Health Expenditure (OOP/THE) |
|  | Sources of Growth in OOP and Prepayment Funds |
|  | Referral path system |
|  | The costs of dying and time remaining to death |
|  | Inequality indicators (Horizontal & Vertical) |
|  | Out-of-pocket changing rules and indicators |
|  | Differences in health payments among different deciles in urban and rural areas |
|  | Inefficiency of the insurance system |
|  | Having made any out of hospital payments linked with the same admission |
|  | Contingent valuing of health insurance premium |
|  | Failure in the rules of economic evaluation |
|  | Lack of well-organized services by the public sector hospitals and clinics or the health insurance support. |
|  | Lack of preventing the private medical persons to work out of the regulated tariff rules or to ignore the insurance organization rules easily |
|  | Inefficient social health insurance mechanism to reduce the direct payments from households |
|  | Health Financing Distribution Indicators of FFCI |
|  | The growth in expenditure on the health administration |
|  | Medical education policies and service delivery model |
| Macroeconomic Indicators | Growth general inflation rate and exchange rate |
|  | Civil status (Development rate) or Human Development Index |
|  | GDP per capita and the (lagged) government share in GDP |
|  | low productivity of the health sector |
|  | Urbanization rate |
|  | The growth in land traffic fatalities |
|  | Iranian targeted subsidy plan |
|  | Unemployment rate |
|  | Budgeting or budget deficit and budget to support the uninsured |
|  | Illiteracy rate |
|  | GGHE-D as percentage of GDP |
|  | Gross national production (GNP) |
|  | Life expectancy increase |
|  | Inequality conditions of the distribution of the risk of financing |
|  | Increase in Liquidity rate |
|  | National income and national consumption |
|  | Population aging |
|  | Population rate |
|  | Dependency ratio |
|  | Currency price unification policy |
|  | War and conflict and quality indicator for years of war |

### **Supplementary 10. Factors associated with catastrophic health expenditure**

| **No.** | **variable** |
| --- | --- |
| 1 | Budget deficit of the health system |
| 2 | Reducing insurance roof ceiling |
| 3 | Increase private service delivery with high tariffs |
| 4 | Increasing the weight of outpatient services to inpatient services |
| 5 | Poor surveillances and control in health system |
| 6 | Conflict of interests of Iran’s health system decision-makers |
| 7 | Lack of implementation of family physician and referral system in the whole country |
| 8 | Domestic mismanagement of insurance organizations |
| 9 | Lack of a codified mechanism for estimating the diagnostic and treatment services actual costs |
| 10 | Economic sanctions against Iran |
| 11 | The increase in the price of medicine and medical devices due to the increase in the exchange rate and the removal of the preferential currency subsidy |
| 12 | Delay in payment of claims of insurance organizations |
| 13 | Inefficiency of the tax system and Tax-based health financing system |
| 14 | Lack of reliable and transparent electronic information systems |
| 15 | Unbalanced growth of wages in the health system and the profit expectations to make money from healthcare services |

### **Supplementary 11. Average score of key variables**

| No | Factors | STEEP | Impact | | | | Uncertainty | | | |
| --- | --- | --- | --- | --- | --- | --- | --- | --- | --- | --- |
|  |  |  | Median | low | medium | high | Median | low | medium | high |
| 1 | Household size | Social | 2 | 3 | 12 | 5 | 1 | 20 | 0 | 0 |
| 2 | Wealth index (income deciles) | Economic | 3 | 0 | 0 | 20 | 1 | 10 | 8 | 2 |
| 3 | Employment status of the household head | Social | 3 | 0 | 4 | 16 | 1 | 13 | 5 | 2 |
| 4 | Gender of the household head | Social | 2 | 1 | 12 | 7 | 1 | 11 | 9 | 0 |
| 5 | The age of the household head | Social | 2 | 8 | 10 | 2 | 1 | 20 | 0 | 0 |
| 6 | Marital status of the household head | Social | 1 | 17 | 3 | 0 | 1 | 20 | 0 | 0 |
| 7 | Gender of patients | Social | 1 | 20 | 0 | 0 | 1 | 20 | 0 | 0 |
| 8 | Education level of the household head | Social | 2 | 1 | 12 | 7 | 1 | 11 | 9 | 0 |
| 9 | Place of residence (urban, rural, remote areas) | Social | 2 | 1 | 12 | 7 | 1 | 11 | 9 | 0 |
| 10 | Homeownership | Economic | 2 | 2 | 12 | 6 | 1 | 18 | 2 | 0 |
| 11 | Household economic status (Q1 vs. Q5) | Economic | 3 | 1 | 9 | 10 | 1 | 18 | 2 | 0 |
| 12 | Dependency ratio | Social | 3 | 2 | 8 | 10 | 1 | 17 | 3 | 0 |
| 13 | Having elderly member (> 60) in Household | Social | 3 | 0 | 8 | 12 | 1 | 10 | 7 | 3 |
| 14 | Population aging | Environmental | 3 | 1 | 9 | 10 | 1 | 16 | 4 | 0 |
| 15 | Inequality distribution of income, education, skills, jobs, opportunities, physician, specialized manpower, health expenditures, and expectations | Political | 2 | 8 | 10 | 2 | 1 | 12 | 8 | 0 |
| 16 | Having member with chronic disease and NCDs | Social | 3 | 0 | 2 | 18 | 1 | 10 | 4 | 6 |
| 17 | Using dentistry services by household members | Economic | 3 | 2 | 8 | 10 | 2 | 4 | 12 | 4 |
| 18 | Using inpatient services and the volume of use by HH members and length of stay | Economic | 2 | 8 | 10 | 2 | 3 | 2 | 6 | 12 |
| 19 | Using outpatient services and the volume of use by HH members | Economic | 3 | 0 | 9 | 11 | 2 | 1 | 14 | 5 |
| 20 | Using diagnostic services | Economic | 2 | 2 | 10 | 8 | 1 | 10 | 6 | 4 |
| 21 | Increasing consumption of expensive high-tech health care services | Social | 3 | 1 | 9 | 10 | 3 | 4 | 7 | 9 |
| 22 | Using private services by HH members | Economic | 3 | 1 | 9 | 10 | 2 | 6 | 9 | 5 |
| 23 | Using Physiotherapy and rehabilitation service | Economic | 2 | 3 | 13 | 4 | 2 | 3 | 12 | 5 |
| 24 | Using medicines and equipment | Economic | 3 | 0 | 4 | 16 | 2 | 8 | 12 | 0 |
| 25 | Informal payments or under-the-counter payment | Economic | 3 | 0 | 9 | 11 | 3 | 3 | 4 | 13 |
| 26 | The medical density | Political | 1 | 12 | 8 | 0 | 1 | 19 | 1 | 0 |
| 27 | Lack of financial protection | Political | 3 | 0 | 2 | 18 | 2 | 6 | 8 | 6 |
| 28 | Payment mechanisms | Economic | 2 | 1 | 10 | 9 | 2 | 5 | 11 | 4 |
| 29 | The health insurance coverage ratio | Political | 3 | 0 | 1 | 19 | 1 | 10 | 7 | 3 |
| 30 | Inequality in access (financial, geographical, and cultural) to healthcare services and safe water | Political | 3 | 2 | 8 | 10 | 2 | 6 | 10 | 4 |
| 31 | Health care tariff growth rate | Economic | 3 | 0 | 3 | 17 | 2 | 5 | 10 | 5 |
| 32 | Physician visits | Economic | 3 | 1 | 6 | 13 | 2 | 9 | 11 | 0 |
| 33 | Induced demand (consumer or supplier) | Political | 3 | 1 | 7 | 12 | 3 | 2 | 9 | 9 |
| 34 | Time of diagnosis | Social | 3 | 0 | 8 | 12 | 2 | 2 | 10 | 8 |
| 35 | GGHE-D as percentage of GDP | Economic | 3 | 2 | 7 | 11 | 2 | 5 | 10 | 5 |
| 36 | Budget deficit of the health system | Economic | 3 | 2 | 6 | 12 | 3 | 5 | 7 | 8 |
| 37 | Reducing insurance roof ceiling | Economic | 3 | 0 | 4 | 16 | 2 | 4 | 10 | 6 |
| 38 | Domestic mismanagement of insurance organizations | Political | 3 | 2 | 8 | 10 | 2 | 9 | 10 | 1 |
| 39 | Increase private service delivery with high tariffs | Economic | 3 | 0 | 4 | 16 | 2 | 4 | 10 | 6 |
| 40 | Increasing the weight of outpatient services to inpatient services | Political | 3 | 2 | 7 | 11 | 2 | 4 | 11 | 5 |
| 41 | The increase in the price of medicine and medical devices due to the increase in the exchange rate and the removal of the preferential currency subsidy | Economic | 3 | 0 | 4 | 16 | 3 | 3 | 4 | 13 |
| 42 | Poor surveillances and control in health system | Political | 3 | 2 | 7 | 11 | 2 | 5 | 14 | 1 |
| 43 | Delay in payment of claims of insurance organizations | Economic | 2 | 3 | 10 | 7 | 2 | 6 | 13 | 1 |
| 44 | Lack of reliable and transparent electronic information systems | Technological | 3 | 3 | 8 | 9 | 2 | 3 | 10 | 7 |
| 45 | Conflict of interests of Iran’s health system decision-makers | Political | 3 | 2 | 6 | 12 | 3 | 5 | 5 | 10 |
| 46 | The profit expectations to make money from healthcare services | Social | 2 | 1 | 10 | 9 | 2 | 3 | 11 | 6 |
| 47 | Inequality conditions of the distribution of the risk of financing | Economic | 2 | 4 | 12 | 6 | 1 | 13 | 6 | 1 |
| 48 | Lack of a codified mechanism for estimating the diagnostic and treatment services actual costs | Political | 2 | 2 | 10 | 8 | 2 | 4 | 10 | 6 |
| 49 | Absence of clinical guidelines | Political | 2 | 3 | 13 | 3 | 1 | 12 | 7 | 1 |
| 50 | Reduction of accumulation of insurance resources | Political | 2 | 8 | 9 | 3 | 1 | 12 | 7 | 1 |
| 51 | Inefficiency of the Tax system and Tax-based health financing system | Political | 3 | 3 | 4 | 12 | 2 | 4 | 11 | 5 |
| 52 | Lack of implementation of family physician and referral system in the whole country | Political | 3 | 0 | 8 | 12 | 3 | 3 | 8 | 9 |
| 53 | Economic sanctions against Iran | Environmental | 3 | 0 | 8 | 12 | 3 | 4 | 4 | 12 |
| 54 | Inflation rate in health sector | Economic | 3 | 0 | 7 | 13 | 3 | 3 | 8 | 9 |
| 55 | GDP per capita and the (lagged) government share in GDP | Economic | 1 | 13 | 7 | 0 | 1 | 13 | 7 | 0 |
| 56 | Households’ Willingness to Pay for Health Services | Social | 1 | 16 | 4 | 0 | 1 | 17 | 3 | 0 |
| 57 | Iranian targeted subsidy plan | Environmental | 1 | 15 | 5 | 0 | 2 | 9 | 11 | 0 |
| 58 | Lifestyle pattern and self-care behavior | Social | 2 | 9 | 11 | 0 | 1 | 17 | 3 | 0 |
| 59 | Life expectancy increase | Social | 2 | 1 | 10 | 9 | 1 | 11 | 8 | 1 |
| 60 | Disease outbreaks and pandemics | Environmental | 2 | 2 | 12 | 6 | 2 | 4 | 10 | 6 |
| 61 | Increase in Liquidity rate | Economic | 2 | 2 | 10 | 8 | 1 | 11 | 8 | 1 |
| 62 | Medical education policies and service delivery model | Political | 2 | 8 | 9 | 3 | 1 | 12 | 7 | 1 |
| 63 | The costs of dying and time remaining to death | Economic | 2 | 9 | 10 | 1 | 2 | 7 | 9 | 4 |
| 64 | Low productivity of the health sector | Economic | 3 | 1 | 7 | 12 | 2 | 9 | 10 | 1 |
| 65 | The growth in expenditure on the health administration | Economic | 3 | 1 | 7 | 12 | 2 | 9 | 10 | 1 |

Following is the priority wise ranking of various scenario drivers:

• High Priority: C18, C21, C25, C33, C36, C41, C44, C45, C52, C53, C54, C17, C19, C22, C24, C27, C30, C31, C32, C34, C35, C37, C38, C39, C40, C42, C51, C64, C65.

• Medium Priority: C23, C28, C43, C46, C48, C60, C63, C2, C3, C11, C12, C13, C14, C16, C29.

• Low Priority: C6, C7, C26, C55, C56, C57, C1, C4, C5, C8, C9, C10, C15, C20, C47, C49, C50, C58, C59, C61, C62.

### **Supplementary 12. Wilson Matrix used to prioritize scenario driver** **[41, 28].**

| Uncertainty | High |  | C_18_ | High Priority  C_21,_ C_25,_ C_33,_ C_36,_ C_41, ,_ C_44,_ C_45,_ C_52,_ C_53,_ C_54,_ |
| --- | --- | --- | --- | --- |
|  | Medium | C_57_ | C_23,_ C_28,_ C_43,_ C_46,_ C_48,_ C_60,_ C_63_  Medium Priority | C_17,_ C_19,_ C_22,_ C_24,_ C_27,_ C_30,_ C_31,_ C_32,_ C_34,_ C_35,_ C_37,_ C_38,_ C_39,_ C_40,_ C_42,_ C_51,_ C_64,_ C_65_ |
|  | Low | Low Priority  C_6,_ C_7,_ C_26,_ C_55,_ C_56,_ | C_1_, C_4_, C_5,_ C_8,_ C_9,_ C_10,_ C_15,_ C_20,_ C_47,_ C_49,_ C_50,_ C_58,_ C_59,_ C_61,_ C_62_ | C_2,_ C_3,_ C_11,_ C_12,_ C_13,_ C_14,_ C_16,_ C_29,_ |
|  |  | Low | Medium | High |
|  | Potential Impact | | | |

### **Supplementary 13. Completed Cross Impact Matrix of key factors**

| 0 | C17 | C18 | C19 | C21 | C22 | C24 | C25 | C27 | C30 | C31 | C32 | C33 | C34 | C35 | C36 | C37 | C38 | C39 | C40 | C41 | C42 | C44 | C45 | C51 | C52 | C53 | C54 | C64 | C65 |
| --- | --- | --- | --- | --- | --- | --- | --- | --- | --- | --- | --- | --- | --- | --- | --- | --- | --- | --- | --- | --- | --- | --- | --- | --- | --- | --- | --- | --- | --- |
| C17 | 0 | 0 | 1 | 2 | 1 | 1 | 1 | 0 | 0 | 0 | 0 | 0 | 0 | 0 | 0 | 0 | 0 | 0 | 1 | 0 | 0 | 0 | 0 | 0 | 0 | 0 | 0 | 0 | 0 |
| C18 | 0 | 0 | 0 | 1 | 1 | 1 | 1 | 0 | 0 | 0 | 0 | 0 | 0 | 0 | 0 | 0 | 0 | 0 | 0 | 0 | 0 | 0 | 0 | 0 | 0 | 0 | 0 | 0 | 0 |
| C19 | 1 | 0 | 0 | 1 | 1 | 1 | 1 | 0 | 0 | 0 | 1 | 1 | 0 | 0 | 0 | 0 | 0 | 1 | 2 | 0 | 0 | 0 | 0 | 0 | 0 | 0 | 0 | 0 | 0 |
| C21 | 1 | 1 | 1 | 0 | 1 | 1 | 0 | 0 | 0 | 0 | 0 | 1 | 0 | 0 | 0 | 0 | 0 | 0 | 0 | 0 | 0 | 0 | 0 | 0 | 0 | 0 | 1 | 0 | 0 |
| C22 | 0 | 0 | 2 | 1 | 0 | 1 | 1 | 0 | 0 | 0 | 1 | 1 | 0 | 0 | 0 | 0 | 0 | 2 | 1 | 0 | 0 | 0 | 0 | 0 | 0 | 0 | 0 | 0 | 0 |
| C24 | 0 | 0 | 0 | 1 | 0 | 0 | 1 | 0 | 0 | 1 | 0 | 2 | 0 | 0 | 0 | 0 | 0 | 0 | 1 | 0 | 0 | 0 | 0 | 0 | 0 | 0 | 1 | 0 | 0 |
| C25 | 0 | 0 | 1 | 0 | 1 | 0 | 0 | 0 | 0 | 1 | 1 | 1 | 0 | 0 | 0 | 0 | 0 | 1 | 1 | 0 | 0 | 1 | 1 | 0 | 0 | 1 | 1 | 1 | 0 |
| C27 | 0 | 0 | 0 | 0 | 0 | 0 | 0 | 0 | 3 | 0 | 0 | 0 | 0 | 0 | 0 | 0 | 1 | 0 | 0 | 0 | 0 | 0 | 0 | 0 | 0 | 0 | 0 | 0 | 0 |
| C30 | 1 | 1 | 1 | 1 | 1 | 1 | 0 | 1 | 0 | 0 | 0 | 0 | 0 | 0 | 0 | 0 | 0 | 0 | 0 | 0 | 0 | 0 | 0 | 0 | 1 | 0 | 0 | 0 | 0 |
| C31 | 1 | 1 | 1 | 1 | 1 | 1 | 0 | 0 | 1 | 0 | 1 | 1 | 0 | 0 | 0 | 0 | 0 | 0 | 0 | 0 | 0 | 0 | 0 | 0 | 0 | 1 | 0 | 0 | 0 |
| C32 | 1 | 0 | 1 | 0 | 1 | 0 | 2 | 0 | 0 | 1 | 0 | 2 | 0 | 0 | 0 | 0 | 0 | 0 | 0 | 0 | 0 | 0 | 0 | 0 | 0 | 1 | 0 | 0 | 0 |
| C33 | 0 | 0 | 0 | 1 | 0 | 1 | 1 | 0 | 1 | 1 | 1 | 0 | 0 | 0 | 0 | 0 | 0 | 1 | 1 | 0 | 0 | 1 | 1 | 0 | 0 | 0 | 0 | 0 | 0 |
| C34 | 1 | 2 | 0 | 3 | 0 | 1 | 0 | 0 | 0 | 0 | 0 | 0 | 0 | 0 | 0 | 0 | 0 | 0 | 0 | 0 | 0 | 1 | 0 | 0 | 1 | 0 | 0 | 0 | 0 |
| C35 | 0 | 0 | 0 | 0 | 0 | 0 | 0 | 0 | 0 | 0 | 0 | 0 | 0 | 0 | 2 | 2 | 0 | 0 | 0 | 0 | 0 | 0 | 0 | 0 | 0 | 1 | 0 | 0 | 0 |
| C36 | 0 | 0 | 0 | 0 | 0 | 0 | 1 | 2 | 0 | 1 | 1 | 1 | 0 | 0 | 0 | 2 | 1 | 1 | 0 | 0 | 0 | 0 | 1 | 0 | 1 | 1 | 0 | 0 | 0 |
| C37 | 0 | 0 | 0 | 0 | 0 | 0 | 0 | 3 | 0 | 0 | 0 | 0 | 0 | 0 | 0 | 0 | 2 | 1 | 1 | 0 | 0 | 0 | 1 | 0 | 1 | 1 | 0 | 0 | 0 |
| C38 | 0 | 0 | 0 | 0 | 0 | 0 | 0 | 2 | 0 | 0 | 1 | 0 | 0 | 0 | 0 | 3 | 0 | 0 | 0 | 0 | 0 | 1 | 0 | 0 | 2 | 0 | 0 | 0 | 0 |
| C39 | 0 | 0 | 0 | 1 | 0 | 0 | 2 | 1 | 0 | 1 | 2 | 1 | 0 | 0 | 0 | 0 | 0 | 0 | 0 | 0 | 0 | 0 | 1 | 0 | 0 | 1 | 0 | 0 | 0 |
| C40 | 1 | 0 | 1 | 1 | 0 | 0 | 1 | 0 | 0 | 1 | 0 | 1 | 0 | 0 | 0 | 0 | 0 | 0 | 0 | 0 | 0 | 0 | 0 | 0 | 1 | 1 | 0 | 0 | 0 |
| C41 | 0 | 0 | 0 | 0 | 0 | 0 | 1 | 1 | 0 | 1 | 0 | 1 | 0 | 0 | 0 | 0 | 0 | 0 | 0 | 0 | 0 | 1 | 1 | 0 | 0 | 1 | 2 | 0 | 0 |
| C42 | 0 | 0 | 0 | 1 | 0 | 0 | 1 | 0 | 0 | 1 | 0 | 1 | 0 | 0 | 0 | 0 | 1 | 0 | 0 | 1 | 0 | 1 | 1 | 0 | 2 | 0 | 1 | 0 | 0 |
| C44 | 0 | 0 | 0 | 0 | 0 | 0 | 1 | 0 | 0 | 0 | 0 | 1 | 1 | 0 | 0 | 0 | 1 | 0 | 0 | 2 | 1 | 0 | 1 | 0 | 2 | 0 | 0 | 0 | 0 |
| C45 | 0 | 1 | 0 | 0 | 0 | 0 | 0 | 1 | 0 | 0 | 0 | 1 | 0 | 1 | 2 | 0 | 1 | 0 | 0 | 1 | 1 | 1 | 0 | 0 | 2 | 0 | 0 | 0 | 0 |
| C51 | 0 | 0 | 0 | 0 | 0 | 0 | 0 | 1 | 0 | 0 | 0 | 0 | 0 | 0 | 3 | 0 | 0 | 0 | 0 | 1 | 0 | 0 | 1 | 0 | 0 | 1 | 0 | 0 | 0 |
| C52 | 0 | 2 | 0 | 0 | 0 | 0 | 0 | 1 | 0 | 0 | 0 | 1 | 1 | 0 | 0 | 0 | 0 | 0 | 0 | 0 | 0 | 1 | 1 | 0 | 0 | 0 | 1 | 1 | 1 |
| C53 | 0 | 0 | 0 | 0 | 0 | 0 | 0 | 0 | 0 | 0 | 0 | 0 | 0 | 2 | 2 | 0 | 0 | 0 | 0 | 3 | 0 | 2 | 1 | 1 | 1 | 0 | 1 | 0 | 0 |
| C54 | 0 | 0 | 0 | 0 | 0 | 0 | 0 | 0 | 0 | 0 | 0 | 0 | 0 | 0 | 3 | 0 | 0 | 0 | 0 | 1 | 0 | 1 | 1 | 0 | 1 | 1 | 0 | 1 | 2 |
| C64 | 0 | 0 | 0 | 0 | 0 | 0 | 0 | 0 | 0 | 0 | 0 | 0 | 0 | 0 | 1 | 0 | 0 | 0 | 0 | 0 | 0 | 1 | 1 | 0 | 0 | 1 | 1 | 0 | 1 |
| C65 | 0 | 0 | 0 | 0 | 0 | 0 | 0 | 0 | 0 | 0 | 0 | 0 | 0 | 0 | 2 | 0 | 0 | 0 | 0 | 0 | 0 | 1 | 1 | 0 | 0 | 1 | 2 | 0 | 0 |

### **Supplementary 14. Potential Direct Influences Matrix.**

| Indicator | Value |
| --- | --- |
| Matrix size | 29 |
| Number of iterations | 2 |
| Number of zeros | 624 |
| Number of ones | 180 |
| Number of twos | 30 |
| Number of threes | 7 |
| Number of P | 0 |
| Total | 217 |
| Fillrate | 25.80262% |

### **Supplementary 15. The Matrix of Direct Effects of Factors**

| **No** | **Variable** | **Total number of rows** | **Total number of columns** |
| --- | --- | --- | --- |
| 1 | Using dentistry services by household members | 7 | 7 |
| 2 | Using inpatient services and the volume of use by HH members and length of stay | 4 | 8 |
| 3 | Using outpatient services and the volume of use by HH members | 10 | 9 |
| 4 | Increasing consumption of expensive high-tech health care services | 11 | 15 |
| 5 | Using private services by HH members | 10 | 8 |
| 6 | Using medicines and equipment | 7 | 9 |
| 7 | Informal payments or under-the-counter payment | 12 | 15 |
| 8 | Lack of financial protection | 4 | 13 |
| 9 | Inequality in accessr | 8 | 6 |
| 10 | Health care tariff growth rate | 10 | 9 |
| 11 | Physician visits | 9 | 9 |
| 12 | Induced demand | 10 | 17 |
| 13 | Time of diagnosis | 9 | 3 |
| 14 | GGHE-D as percentage of GDP | 5 | 3 |
| 15 | Reducing the budget of the health system | 13 | 15 |
| 16 | Reducing insurance roof ceiling | 10 | 7 |
| 17 | Domestic mismanagement of insurance organizations | 9 | 7 |
| 18 | Increase private service delivery with high tariffs | 10 | 7 |
| 19 | Increasing the weight of outpatient services to inpatient services | 7 | 8 |
| 20 | The increase in the price of medicine and medical devices due to the increase in the exchange rate and the removal of the preferred currency | 9 | 10 |
| 21 | Poor surveillances and control in health system | 11 | 2 |
| 22 | Lack of reliable and transparent electronic information systems | 10 | 13 |
| 23 | Conflict of interests of Iran's health system decision-makers | 12 | 14 |
| 24 | Inefficiency of the Tax system and Tax-based health financing system | 7 | 1 |
| 25 | Lack of establishment of family physician and referral system in the whole country | 10 | 15 |
| 26 | Economic sanctions against Iran | 13 | 13 |
| 27 | Inflation rate in health sector | 11 | 11 |
| 28 | Low productivity of the health sector | 6 | 3 |
| 29 | The growth in expenditure on the health administration | 7 | 4 |
|  | Totals | 261 | 261 |

### **Supplementary 16. The Matrix of indirect Effects of Factors**

| **No** | **Variable** | **Total number of rows** | **Total number of columns** |
| --- | --- | --- | --- |
| 1 | Using dentistry services by household members | 638 | 588 |
| 2 | Using inpatient services and the volume of use by HH members and length of stay | 380 | 774 |
| 3 | Using outpatient services and the volume of use by HH members | 886 | 813 |
| 4 | Increasing consumption of expensive high-tech health care services | 894 | 1114 |
| 5 | Using private services by HH members | 931 | 741 |
| 6 | Using medicines and equipment | 680 | 806 |
| 7 | Informal payments or under-the-counter payment | 1151 | 1356 |
| 8 | Lack of financial protection | 266 | 1075 |
| 9 | Inequality in accessr | 570 | 735 |
| 10 | Health care tariff growth rate | 836 | 917 |
| 11 | Physician visits | 894 | 912 |
| 12 | Induced demand | 914 | 1655 |
| 13 | Time of diagnosis | 682 | 368 |
| 14 | GGHE-D as percentage of GDP | 533 | 337 |
| 15 | Reducing the budget of the health system | 1114 | 1010 |
| 16 | Reducing insurance roof ceiling | 736 | 507 |
| 17 | Domestic mismanagement of insurance organizations | 661 | 626 |
| 18 | Increase private service delivery with high tariffs | 968 | 719 |
| 19 | Increasing the weight of outpatient services to inpatient services | 667 | 760 |
| 20 | The increase in the price of medicine and medical devices due to the increase in the exchange rate and the removal of the preferred currency | 894 | 939 |
| 21 | Poor surveillances and control in health system | 1044 | 260 |
| 22 | Lack of reliable and transparent electronic information systems | 928 | 1216 |
| 23 | Conflict of interests of Iran's health system decision-makers | 1013 | 1250 |
| 24 | Inefficiency of the Tax system and Tax-based health financing system | 726 | 102 |
| 25 | Lack of establishment of family physician and referral system in the whole country | 755 | 1286 |
| 26 | Economic sanctions against Iran | 1220 | 997 |
| 27 | Inflation rate in health sector | 1103 | 927 |
| 28 | Low productivity of the health sector | 651 | 373 |
| 29 | The growth in expenditure on the health administration | 804 | 376 |
|  | Totals | 261 | 261 |

### **Supplementary 17. Direct influence and dependence of variables**

| **Rank** | **Role** | **Label** | **Variable** | MDI | |
| --- | --- | --- | --- | --- | --- |
|  |  |  |  | Direct influence | Direct dependence |
| 1 | Key variables | C36 | Budget deficit of the health system | 498 | 574 |
| 2 |  | C53 | Economic sanctions against Iran | 498 | 498 |
| 3 |  | C25 | Informal payments or under-the-counter payment | 459 | 574 |
| 4 |  | C45 | Conflict of interests of Iran’s health system decision-makers | 459 | 536 |
| 5 |  | C21 | Increasing consumption of expensive high-tech health care services | 421 | 574 |
| 6 |  | C54 | Inflation rate in health sector | 421 | 421 |
| 7 |  | C33 | Induced demand (consumer or supplier) | 383 | 651 |
| 8 |  | C44 | Lack of reliable and transparent electronic information systems | 383 | 498 |
| 9 |  | C52 | Lack of implementation of family physician and referral system in the whole country | 383 | 574 |
| 10 |  | C41 | The increase in the price of medicine and medical devices due to the increase in the exchange rate and the removal of the preferential currency subsidy | 344 | 383 |
| 11 | Input | C42 | Poor surveillances and control in health system | 421 | 76 |
| 12 |  | C19 | Using outpatient services and the volume of use by HH members | 383 | 344 |
| 13 |  | C22 | Using private services by HH members | 383 | 306 |
| 14 |  | C31 | Health care tariff growth rate | 383 | 344 |
| 15 |  | C37 | Reducing insurance roof ceiling | 383 | 268 |
| 16 |  | C39 | Increase private service delivery with high tariffs | 383 | 268 |
| 17 |  | C32 | Physician visits | 344 | 344 |
| 18 |  | C34 | Time of diagnosis | 344 | 114 |
| 19 |  | C38 | Domestic mismanagement of insurance organizations | 344 | 268 |
| 20 | Excluded | C30 | Inequality in access (financial, geographical, and cultural) to healthcare services and safe water | 306 | 229 |
| 21 |  | C17 | Using dentistry services by household members | 268 | 268 |
| 22 | Clustered | C24 | Using medicines and equipment | 268 | 344 |
| 23 | Excluded | C40 | Increasing the weight of outpatient services to inpatient services | 268 | 306 |
| 24 |  | C51 | Inefficiency of the Tax system and Tax-based health financing system | 268 | 38 |
| 25 |  | C65 | The growth in expenditure on the health administration | 268 | 153 |
| 26 |  | C64 | Low productivity of the health sector | 229 | 114 |
| 27 |  | C35 | GGHE-D as percentage of GDP | 191 | 114 |
| 28 |  | C18 | Using inpatient services and the volume of use by HH members and length of stay | 153 | 306 |
| 29 | Dependent | C27 | Lack of financial protection | 153 | 498 |
